# TNF-α Acts as an Immunoregulator in the Mouse Brain by Reducing the Incidence of Severe Disease Following Japanese Encephalitis Virus Infection

**DOI:** 10.1371/journal.pone.0071643

**Published:** 2013-08-05

**Authors:** Daisuke Hayasaka, Kenji Shirai, Kotaro Aoki, Noriyo Nagata, Dash Sima Simantini, Kazutaka Kitaura, Yuki Takamatsu, Ernest Gould, Ryuji Suzuki, Kouichi Morita

**Affiliations:** 1 Department of Virology, Institute of Tropical Medicine, GCOE program, Leading Graduate School Program, Nagasaki University, Nagasaki, Nagasaki, Japan; 2 Clinical Research Center, National Hospital Organization Sagamihara Hospital, Sagamihara, Kanagawa, Japan.; 3 Department of Pathology, National Institute of Infectious Diseases Musashimurayama, Tokyo, Japan.; 4 UMR 190 Emergence des Pathologies Virales, Aix-Marseille Univ & Institut de Recherche pour le Développement, Marseille, France and NERC CEH, Wallingford, United Kingdom

## Abstract

Japanese encephalitis virus (JEV) causes acute central nervous system (CNS) disease in humans, in whom the clinical symptoms vary from febrile illness to meningitis and encephalitis. However, the mechanism of severe encephalitis has not been fully elucidated. In this study, using a mouse model, we investigated the pathogenetic mechanisms that correlate with fatal JEV infection. Following extraneural infection with the JaOArS982 strain of JEV, infected mice exhibited clinical signs ranging from mild to fatal outcome. Comparison of the pathogenetic response between severe and mild cases of JaOArS982-infected mice revealed increased levels of TNF-α in the brains of severe cases. However, unexpectedly, the mortality rate of TNF-α KO mice was significantly increased compared with that of WT mice, indicating that TNF-α plays a protective role against fatal infection. Interestingly, there were no significant differences of viral load in the CNS between WT and TNF-α KO mice. However, exaggerated inflammatory responses were observed in the CNS of TNF-α KO mice. Although these observations were also obtained in IL-10 KO mice, the mortality and enhanced inflammatory responses were more pronounced in TNF-α KO mice. Our findings therefore provide the first evidence that TNF-α has an immunoregulatory effect on pro-inflammatory cytokines in the CNS during JEV infection and consequently protects the animals from fatal disease. Thus, we propose that the increased level of TNF-α in severe cases was the result of severe disease, and secondly that immunopathological effects contribute to severe neuronal degeneration resulting in fatal disease. In future, further elucidation of the immunoregulatory mechanism of TNF-α will be an important priority to enable the development of effective treatment strategies for Japanese encephalitis.

## Introduction

Japanese encephalitis virus (JEV), which belongs to the genus Flavivirus in the family *Flaviviridae*, is a causative agent of acute central nervous system (CNS) disease in humans and domestic animals [1]. Pigs and birds are amplifiers or reservoir hosts of JEV in the environment, providing a source of virus for blood feeding *Culex* spp. mosquitoes [1] which may subsequently feed on and infect humans. Japanese encephalitis (JE) is a major public health issue in Asia and the Asia-Pacific region [1,2]. Annually, 30,000-50,000 cases and 10,000-15,000 deaths are reported and more than 50% of survivors may suffer from neurological disability [2].

Human infections are largely subclinical with a rate varying from 1:25 to 1:1000 [3,4]. The clinical symptoms vary from mild to severe disease including a non-specific febrile illness, meningitis, encephalitis and meningoencephalitis, the latter being observed in the most severe cases [4–6]. . Following an incubation period of 6-16 days, patients may develop fever, headache, vomiting, rigor and nausea [1,4]. Subsequently, encephalitic cases develop neurological symptoms including seizure, tremor, photophobia, decreased sensorium, generalized and localized paresis, movement disorder [4,6]. Signs of meningeal irritation such as neck stiffness may be present. These clinical features are not unique to JE, thus, laboratory diagnosis is required to distinguish it from other neurological disorders.

The variety of disease symptoms and the prognosis in JE cases appears to be dependent on complex interactions between viral and host factors [4]. In particular, host factors appear to be important determinants of disease severity and a number of specific proinflammatory cytokines and chemokines are observed in severe JE cases [7]. For example, it has been demonstrated that increased levels of TNF-α in cerebrospinal fluid (CSF) and serum correlated with cases of severe disease [8]. However, how these biological cytokines and chemokines contribute to the severe disease has not been fully elucidated. Therefore, understanding the mechanism of the specific host response in severe cases is an important priority to develop a specific treatment for the infectious disease.

The laboratory mouse model is commonly employed to study the CNS pathology induced by encephalitic flaviviruses [9–11]. In common with human cases, mice develop relatively similar neurological dysfunction and the pathologic changes in infected mouse brains are similar to those observed in human cases [6,9]. Although extraneural infection frequently does not result in detectable viremia or virus burden in mice, it is believed that initial virus replication occurs in dendritic cells (DCs) such as Langerhans cells at the site of infection, and the infected DCs migrate to draining lymph nodes [6]. Virus then invades the CNS and hosts develop CNS disease, although the mechanism by which the blood–brain-barrier is crossed is not completely understood [12–18].

CNS pathology is the consequence of viral infection of the affected cells and the resulting inflammatory responses in the CNS. Flavivirus variants may induce different degrees of pathology, however, the host immune response is likely to be a more critical determinant of clinical outcome [19]. Inflammatory responses mainly contribute to virus clearance and recovery from fatal disease. For example, CD8^+^ T cells are reported to have an important function in controlling virus infection [20–25], although one report showed only a subsidiary contribution of CD8^+^ T cells in JEV infection [26]. On the other hand, in recent studies it was suggested that immunopathological mechanisms may contribute to the severity of outcome following some encephalitic flavivirus infections [19,27–30]. For example, it was reported that CD8^+^ T cell function enhances pathogenicity during WNV and MVEV infections [29,30]. Furthermore, in Tick-borne encephalitis virus (TBEV)-infected mice the inflammatory response was reported to contribute significantly to the fatal outcome [28].

Microglia are the resident macrophages in the brain and are activated in response to a number of different pathological states [31]. Following JEV infection, activated microglia play a significant role in the development of pathology by producing pro-inflammatory cytokines such as IL-1, IL-6 and TNF-α [32,33]. Although these pro-inflammatory cytokines have dual roles, acting both as protectors and degenerators of neurons [31], TNF-α is believed to be one of the key factors that mediate immunopathology in the CNS during encephalitic flavivirus infection. For example, it was suggested that TNF-α directly mediates neuronal apoptosis by the engagement of TNF receptor 1 (TNFR1), the TNFR-associated death domain (TRADD) and neuronal death contributes to glial activation and subsequent neuroinflammation [31,34,35]. It was also shown that TNF-α and IL-1β mediate RANTES gene expression for the recruitment of immune cells and glutamate released by JEV-infected microglia, involves TNF-α signaling and contributes to neuronal death [36,37]. On the other hand, other studies have shown that TNF-α has a protective role against WNV infections and restricts WNV pathogenesis by promoting trafficking of mononuclear leukocytes into the CNS [38,39]. Furthermore, neuronal TNF-α expression during WNV encephalitis may be an adaptive response to diminish CXCL10-induced death [40]. At this stage of our knowledge, therefore, the precise role of the TNF-α response in encephalitic flaviviral pathogenesis remains to be clarified.

Immunomodulatory cytokines also affect disease outcome of encephalitic flavivirus infection. IL-10 is reported to have an effect on immunoregulation [41]. It was suggested that IL-10 mediates protection from acute encephalitis and plays a central role in determining the clinical outcome of JEV infection [42]. Insufficient anti-inflammatory cytokine production of IL-4 and IL-10 in the brain is associated with increased tissue pathology [43]. IL-10 displays a neuroprotective function during JEV infection and regulates deleterious effects of proinflammatory cytokines [44]. Furthermore, an experiment using IL-10 KO mice showed that IL-10 signaling plays a negative role in immunity against WNV infection and blockade of IL-10 signaling helps to control viral infection [45]. Thus, the precise role of the IL-10 response following encephalitic flavivirus infection also remains to be resolved.

In general, evaluation of virus pathogenicity and virulence in mouse models utilizes either the subcutaneous or intradermal route of infection. This is considered to be a reproducible model of natural human infections following the bite of an infected mosquito or tick and in the past, death was used as an index of pathogenesis [46]. However, it is known that peripheral infections with some strains of encephalitic flaviviruses do not exhibit normal dose response curves based on mortality. Although this was first reported in the 1940’s [47], the reason for these apparent discrepancies were not fully understood. We previously showed that the Oshima strain of TBEV caused dose independent mortality and the fatality rate did not increase more than 50% with increasing virus challenge doses from 10^2^ to 10^6^ plaque forming unit (pfu) [48]. In our study of TBEV, we suggested that the variation of fatal outcome in individual mice appeared to be due to variation in individual host responses [48].

The purpose of this study was to investigate the host factors that influence disease severity following JEV infection in a mouse model. In particular, we focused on the variation of disease outcome in individual mice following extraneural infection with JEV. We first compared the pathogenicity of two JEV strains, which cause either dose-dependent or dose-independent mortality responses. We next compared severe or mild cases of mice infected with JEV exhibiting dose-independent mortality and investigated the specific host responses such as TNF-α and IL-10 expression in the CNS. We also examined the roles of the specific cytokines observed in severe cases using appropriate knockout (KO) mice.

## Materials and Methods

### Ethics statement

The animal experiments were performed in accordance with the recommendations in the Fundamental Guidelines for Proper Conduct of Animal Experiment and Related Activities in Academic Research Institutions under the jurisdiction of the Ministry of Education, Culture, Sports, Science and Technology. The experimental protocols were approved by the Animal Care and Use Committee of the Nagasaki University (approval number: 091130-2-7/0912080807-7).

### Virus and cells

The JaTH160 strain of JEV was kindly provided by Tomohiko Takasaki, National Institute of Infectious Disease, Japan. Stocks of JEV JaOArS982 and JaTH160 viruses were obtained from cell culture medium of baby hamster kidney (BHK) cells infected with viruses previously prepared in suckling mouse brains [49]. The BHK cells were maintained in Eagle’s Minimal Essential Medium (EMEM; Nissui Pharmaceutical Co.) containing 8% fetal calf serum (FCS) and antibiotics.

### Mice

C57BL/6j (B6) mice were purchased from Japan SLC Corporation. B6 background IL-10-/- mice were purchased from Jackson Laboratory, USA [50]. TNF -/- mice were kindly provided by Yoichiro Iwakura, Research Institute for Biomedical Sciences, Tokyo University of Science [51]. These mice were mated in the facility of Nagasaki University. Five to six week old mice were subcutaneously inoculated with a range of 10^0^-10^6^ pfu of JEV diluted in EMEM containing 2% FCS. Mock infected mice were inoculated with EMEM from the supernatant medium of BHK cells. Mice were weighed daily and observed for clinical signs including behavioral symptoms and signs of paralysis. Thirteen days post infection (pi), dying and recovering mice were distinguished by the degree of weight ratio, and namely mice exhibiting more than 25% or less than 10% weight loss were recognized as dying or recovering mice, respectively.

### Virus titration in tissues

Following subcutaneous inoculation with 10^4^ pfu of JEV, mice were euthanized and blood, spleen, brain and spinal cord were collected following perfusion with cold phosphate-buffered saline (PBS). Brains were dissected to provide four separate fractions, ie the brain cortex, thalamus, cerebellum and brainstem. Until they were used, these tissues were stored at -80° C. Each tissue was homogenized in ten volumes of PBS containing 10% FCS and diluted with EMEM with 2% FCS. Virus titers were determined by plaque forming assays using BHK cells and were expressed as pfu/g tissue [48].

### Quantitative estimation of the expression of inflammatory cytokines in brains and spleens

Mouse brains and spleens were collected after perfusion with cold PBS. Freshly isolated brains and spleens were immediately immersed in RNAlater (Ambion). Total RNA was extracted using RNeasy Lipid Tissue Mini Kit (Qiagen) according to the manufacturer’s instructions. The expression levels of cytokines were measured by real time-PCR as demonstrated previously [52]. The copy numbers were calculated as a ratio of the copy numbers of internal control glyceraldehyde-3-phosphate dehydrogenate.

### Histopathological examination

Mice inoculated with JEV were anesthetized and perfused with 10% phosphate-buffered formalin. Fixed tissues were routinely embedded in paraffin, sectioned, and stained with hematoxylin and eosin. Immunohistochemical detection of the JEV antigens was performed as described previously [53]. Rabbit polyclonal antibody against E protein was used to detect JEV antigens.

### Determination of virus sequences recovered from severe and mild cases of JaOArS982-infected mice

Brains were collected from four mice in each group of dying and recovering mice at 13 days pi. The brains were homogenized and passaged in BHK cells for 2 days. Viral RNA was extracted from the supernatant medium of the BHK cells using QIAquick PCR Purification Kit (QIAGEN) according to manufacturer’s protocol. Reverse transcription was performed by using Superscript III reverse transcriptase (Invitrogen) and random hexamers. PCR was performed to cover the whole genome sequence using TAKARA Ex *Taq* DNA polymerase (TAKARA BIO Inc.). The cycle sequencing reaction was performed by using BigDye Terminator v 3.1 Cycle Sequencing kit (Life Technologies) and the DNA sequence was determined with Applied Biosystems 3730 DNA Analyzer (Life Technologies).

### Estimations of hormones and cytokine levels in the serum

Serum samples were collected from infected mice. The levels in the serum were measured by using competitive enzyme immunoassay and sandwich enzyme-linked immunosorbent assay kits for corticosterone (AssayPro), TNF-α and IL-10, IL-12 (Endogen) according to the manufacturer’s instructions.

### Recovery of leukocytes from brain and thymus

Recovery of leukocytes was performed by applying previously described methods [22,54]. Briefly, after perfusion with cold PBS, brains and thymus were removed and placed on ice in RPMI containing 5% FCS (Nissui Pharmaceutical Co.). Brains were strained and homogenized gently with a 70 µm cell strainer (BD Biosciences). After washing with RPMI, the cell suspension was layered onto a 70% and 30% Percoll gradient (GE Healthcare Bio-sciences AB) and centrifuged at 800 × g for 45 min at 23° C. The leukocytes were collected from between the 70% and 30% interface. Thymocytes and splenocytes were also recovered from these mice. Cells were strained with a 70 µm cell strainer (BD Biosciences) and lysed with RBC lysis buffer (Sigma-Aldrich). After washing, cells were resuspended in RPMI medium. Isolated cells were counted and kept on ice until the staining procedure.

### Flow cytometric analysis of cell-surface antigens

Brain leukocytes were washed and blocked with Rat Anti-Mouse CD16/32 (Fc Receptor) (Beckman Coulter) in FACS buffer (PBS containing 0.1% BSA and 0.1% sodium azide). Cells were stained with a mixture of different fluorescent-labeled antibodies directed at surface phenotypic markers, CD45-FITC, F4/80-PE, NK1.1-PerCP-Cy5.5, CD4-PE-Cy7, CD8-APC, CD19-Alexa Fluor 700, CD3e-eFluor 450 (Beckman Coulter) and then fixed with 4% paraformaldehyde overnight. The stained cells were analyzed by Galios™ flow cytometer (Beckman Coulter). Leukocytes were recognized by characteristic size (forward scatter), granularity (side scatter) and CD45 expression. Thymocytes were recognized by their characteristic size and CD4^+^CD8^+^ double positive cells were recognized by the expression of CD4^+^ and CD8^+^.

### Statistical analyses

Kruskal-Wallis test, and Mann Whitney test were used for statistical analysis to assess the significant differences of viral loads, expression levels of cytokines, and numbers of leukocytes. Gehan-Breslow-Wilcoxon Test was performed to assess the survival curves of JEV-infected mice groups. *P* value <0.05 was considered statistically significant.

## Results

### Mortality of JaOArS982- and JaTH160-infected mice

**Figure 1:**
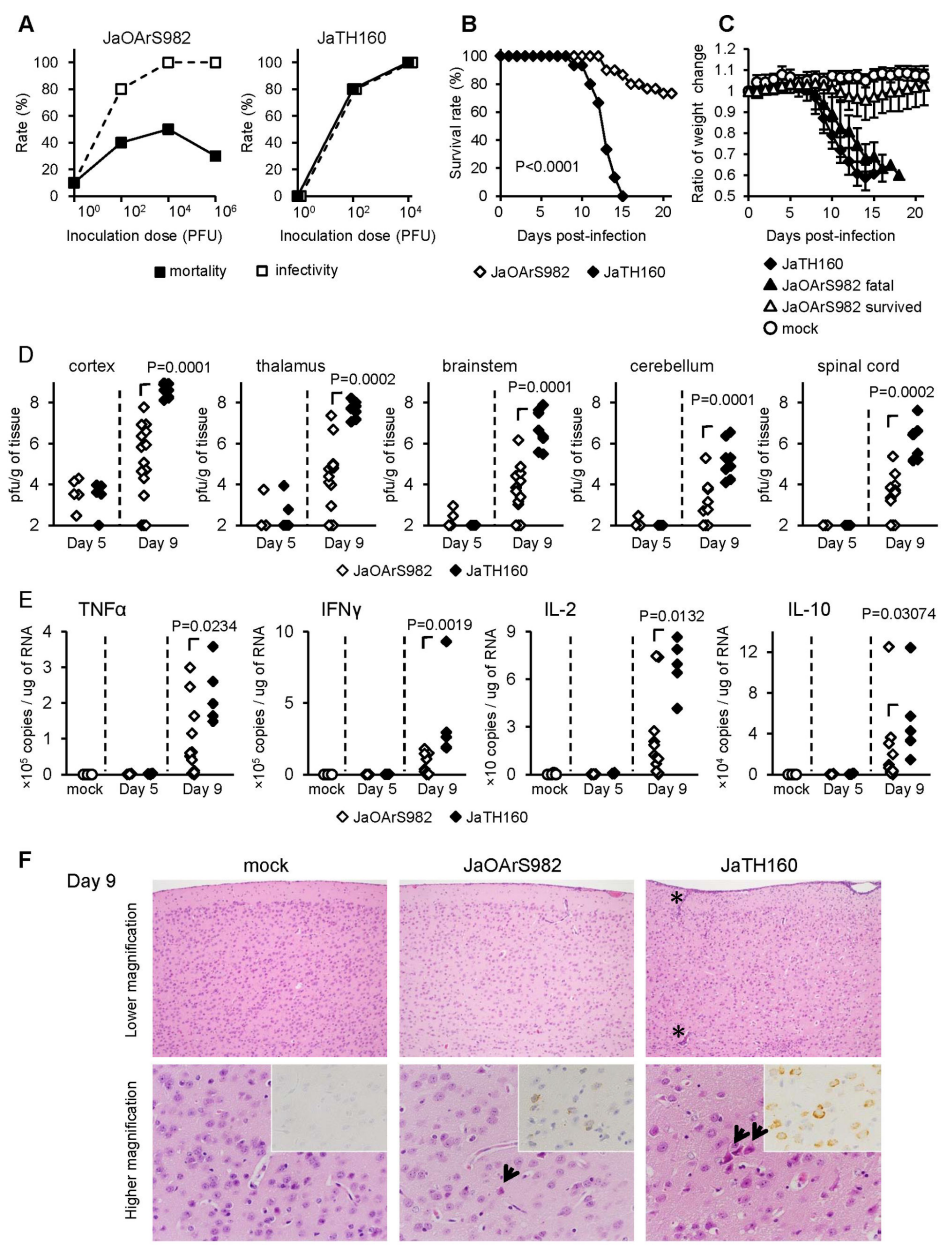
Mortality and pathogenicity of B6 mice subcutaneously infected with JaOArS982 and JaTH160. (A) Mortality and infectivity rates of B6 mice (n=10) following subcutaneous infections with 10^0^, 10^2^, 10^4^ and 10^6^ pfu of JaOArS982 and JaTH160. Mice were recorded for 21 days and no mice died after 21 days. Infectivity was determined by anti-JEV IgG antibody seroconversion for more than 1:1000 of IgG ELISA titer. (B and C) B6 mice were subcutaneously infected with 10^4^ pfu of JaOArS982 (n=30) and JaTH160 (n=15). Survival curves P: Gehan-Breslow-Wilcoxon Test. (C) The averages ratio of weight change of living mice at the time points compared with those of day 0 following subcutaneous infections with 10^4^ pfu of JaOArS982 (n=30) and JaTH160 (n=15). Error bars represent the standard deviations. (D) Viral loads in distinct regions of the CNS following subcutaneous infections with 10^4^ pfu of JaOArS982 (Day 5: n=5, Day 9: n=15) and JaTH160 (Day 5: n=5, Day 9: n=8). P: Mann Whitney test. (E) mRNA levels of TNF-α, IFNγ, IL-2 and IL-10 quantified by real-time PCR in the brain cortex of B6 mice infected with 10^4^ pfu of JaOArS982 (Day 5: n=5, Day 9: n=12), JaTH160 (Day 5: n=5, Day 9: n=5) and mock (n=8). P: Mann Whitney test. (F) Histopathological features of brain cortex in B6 mice infected with 10^4^ pfu of JaOArS982 and JaTH160 at 9 days pi. JEV antigens were detected using E-protein specific JEV antibody (insets). Each experiment represents six and four mice infected with JaOArS982 and JaTH160, respectively. JaOArS982-infected mice showed slight inflammatory infiltration in meninges. In brain cortex, a few degenerated cells were presented (arrow) and were virus antigen-positive cells. In JaTH160-infected mice, severe inflammatory reactions were seen in meninges and perivascular area (asterisks). Many virus antigen-positive cells were detected in degenerated neuronal cells of the cortex (arrows).

In this study, we used inbred B6 mice to minimize the influence of the genetic background of individuals. Subcutaneous infection with JaOArS982 did not lead to a normal dose dependent curve of mortality (Figure 1A). The mortality rate was not significantly increased when challenge doses ranged from 10^2^ to 10^6^ pfu per mouse, although the infectivity in the mice increased sequentially (Figure 1A). On the other hand, JaTH160 infection exhibited a dose dependent mortality curve and the infectivity in the mice was consistent with the mortality (Figure 1A). These observations indicate that individual JaOArS982-infected mice exhibit a variable prognosis independent of virus challenge dose, whereas all JaTH160-infected mice died.

Because a virus challenge dose of 10^4^ pfu of either JaOArS982 or JaTH160 induced 100% infectivity (Figure 1A), this dose was used for all further investigations to compare the pathogenesis. JaOArS982-infected mice did not start to die until 13 days pi and the mean survival time (MST) was 15.5 ± 2.56 days (Figure 1B). Mice that died exhibited generalized clinical signs involving slowness in movement, ataxia, piloerection and anorexia. Continuous weight loss was observed in mice that died, whereas survivors regained weight from 13 to 15 days pi onwards (Figure 1C). On the other hand, JaTH160-infected mice started to die at 9 days pi and all mice had died by 15 days pi (Figure 1B) following continuous weight loss (Figure 1C). MST was 12.8 ± 0.89 days and was significantly shorter than that of JaOArS982-infected mice (Mann Whitney test, P=0.0173). Thus, we hypothesized that the cause of fatal disease was different between JaOArS982- and JaTH160-infected mice.

### Comparison of viral loads and inflammatory responses in the CNS of JaOArS982- and JaTH160-infected mice

Infectious virus was detectable in the brain cortex and thalamus at 5 days pi in both JaOArS982 and JaTH160-infected mice without significant difference in titer (Figure 1D). However, at 9 days pi viral loads of JaTH160-infected mice were significantly higher than those of JaOArS982-infected mice in every region of the brain cortex, thalamus, brainstem, cerebellum and spinal cord (Figure 1D).

It is important to note that viral load in the brain cortex was higher than in other regions of the CNS in both JaOArS982 and JaTH160-infected mice (Figure 1D), indicating that the brain cortex is the main target region for JEV infection. Thus, we next examined the cytokine levels in the brain cortex to compare the immune responses. The levels of TNF-α, IFN-γ, IL-2 and IL-10, but not IL-4 and IL-5 were significantly higher in JaTH160-infected mice than in JaOArS982-infected mice (Figure 1E, Figure S1A). The cytokine levels of TNF-α, IFN-γ, IL-2 and IL-10 were very low or undetectable in mock-infected mice and in infected mice at 5 days pi (Figure 1E). Corresponding to the viral loads, histopathological examination showed that a large number of neurons displayed JEV antigens and severe cuffing was observed in the brain cortex of JaTH160-infected mice at 9 day pi (Figure 1F). JaOArS982-infected mice also exhibited JEV antigen-positive neurons and cuffing, but at lower levels than those observed in JaTH160-infected mice (Figure 1F). Mock-infected mice showed no JEV antigen-positive neurons or inflammatory reactions (Figure 1F). These results confirm that during the early phase of infection, JaTH160-infected mice developed severe encephalitis with extensive neuronal infection which contrasts with the less extensive neuronal infection induced in JaOArS982-infected mice.

Infectious virus was either not detectable or very limited in spleens (data not shown). Interestingly, the levels of TNF-α and IL-2 in spleens were up-regulated in JaOArS982-infected mice at 5 and 9 days pi, however, they were not elevated in JaTH160-infected mice (Figure S1B). The levels of IFN-γ, IL-4, IL-5 and IL-10 were not significantly different between JaOArS982 and JaTH160-infected mice (Figure S1B). These observations suggest that i) inflammatory responses in peripheral organs were different from those in the CNS, and ii) JaTH160 infection induced no significant expression of TNF-α in the spleen.

### JaOArS982 virus quasispecies in the brain do not contribute to the divergence of disease severity

In view of the observation that individual mice displayed different disease progress when infected with JaOArS982 under identical conditions, we attempted to identify specific factors relating to disease severity outcome. Initially, we attempted to discriminate severe and mild disease groups during the observation period by following the progression of weight change of individual mice. We discriminated dying and recovering mice based on whether they showed less than 0.75 or more than 0.90 of the weight ratio at 13 days pi (Figure 1C). It was difficult to predict if mice would survive or die between 0.75 and 0.9 of the designated weight ratio, because within this window both dead mice and survivors were recorded. Having established this defining parameter between severe and mild disease groups, we then attempted to examine specific factors relating to disease severity.

**Figure 2:**
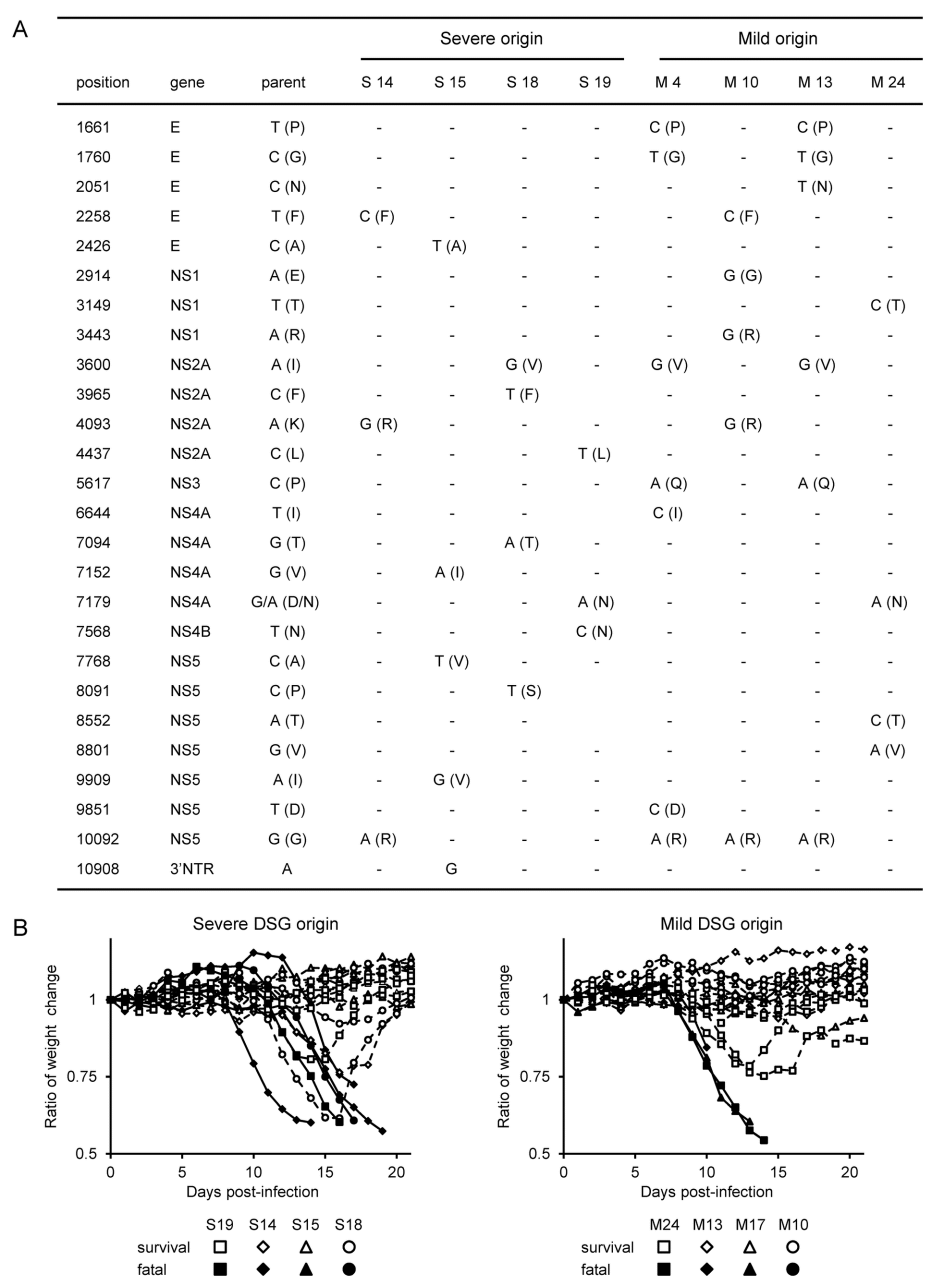
Viral sequences and their potential for fatal infection recovered from JaOArS982-infected mice. (A) Comparison of viral genome sequences (nucleotide and corresponding amino acid – in parentheses) between recovered viruses from brains of severe (S14, S15, S18 and S19) and mild (M4, M10, M13 and M24) cases of JaOArS982-infected B6 mice. (B) Weight changes of B6 mice infected with 10^4^ pfu of recovered viruses from severe (S14, S15, S18 and S19) and mild (M17 replaced to M4, M10, M13 and M24) cases. Five mice in each group were inoculated subcutaneously and observed for 21 days. Closed and open symbols identify mice that died or survived, respectively, during observation period.

We initially considered the possibility that the divergence of disease severity might be due to the selection of quasispecies variants from the JaOArS982 virus population in the CNS. Accordingly, we compared the virus sequences recovered from the brains of dying and recovering mice (Figure 2A). However, no specific virus sequence differences were detected in viruses from either the severe or mild disease severity groups (DSG) (Figure 2A). Furthermore, recovered viruses from either severe or mild DSG exhibited similar mortality patterns to those of the parent JaOArS982 virus (Figure 2B). Noticeably, the viruses recovered from severe DSG mice did not induce 100% lethal infection in subsequent mouse virulence tests (Figure 2B).

These results indicate that quasispecies variants of JaOArS982 did not contribute to the divergence of disease progression observed in all experiments with this virus; thus, other factors such as host response seem most likely to be the determinants of disease severity.

### Comparison of viral loads and inflammatory responses in the CNS of different DSG in JaOArS982-infected mice

**Figure 3:**
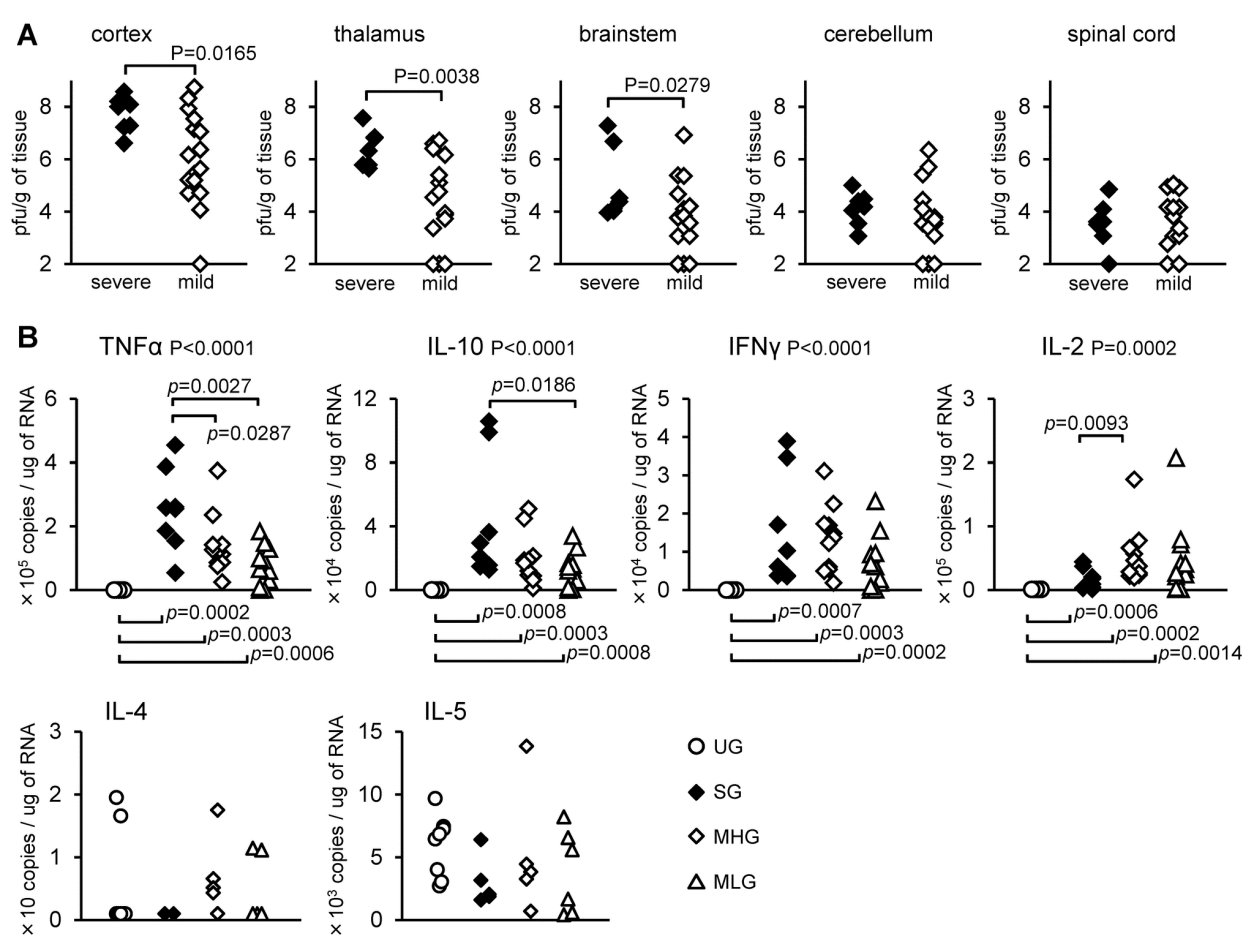
Viral loads and cytokine levels in the brains of severe and mild cases of JaOArS982-infected mice. (A) Viral loads in the CNS of severe (weight loss: <0.75, n=8) and mild (weight loss: >0.90, n=24) cases of JaOArS982-infected B6 mice at 13 days pi. P: Mann Whitney test. (B) mRNA levels of TNF-α, IL-10, IFNγ, IL-2, IL-4 and IL-5 quantified by real-time PCR in the brain cortex of JaOArS982-infected B6 mice at 13 days pi. Uninfected group (U group, n=8), Severe group: S (n=8), Mild group with high viral load of >10^6^ pfu/g of brain tissue: MH (n=11), Mild group with low viral load of <10^6^ pfu/g of brain tissue: ML (n=13). P: Kruskal-Wallis test, *p*: Mann Whitney test.

We next compared the viral loads in the CNS between severe and mild DSG in JaOArS982-infected mice at 13 days pi. Viral loads in brain cortex, thalamus and brainstem but not cerebellum and spinal cord were significantly higher in severe DSG mice than in mild DSG (Figure 3A). However, in the brain cortex, the variance of virus titer in the mild DSG mice ranged from the minimal detection limit to 10^8^ pfu/g of tissue, whereas all mice exhibited more than 10^6^ pfu/g of tissue in the severe DSG (Figure 3A). These results imply that 45.8% (11/24) of mice in the mild DSG produced high viral loads similar to those in the severe DSG. Thus, it is likely that high viral infection alone is not a critical determinant of severe disease and additional factors contribute to the fatal encephalitis.

Therefore, to compare the specific immune responses in severe cases, we further subdivided the mice into three subgroups, severe group (SG), mild group with high viral load (>10^6^ pfu/g of tissue) (MHG) or low viral load (<10^6^ pfu/g of tissue) (MLG), and compared their cytokine levels in the brain cortex (Figure 3B). All groups exhibited increased levels of inflammatory cytokines of TNF-α, IL-10, IFN-γ and IL-2, but not IL-4 and IL-5 in the brain cortex compared with the uninfected group (UG) (Figure 3B). Interestingly, the level of TNF-α in the SG was significantly increased when compared with those in the MHG and MLG (Figure 3B). The level of IL-10 in the SG was also significantly higher than in the MLG (Figure 3B). Although the difference was not significant, the level tended to be higher than that recorded in the MHG (Figure 3B). On the other hand, IFN-γ did not show significant differences between the three groups, and IL-2 levels in the SG were lower than in the MHG (Figure 3B).

**Figure 4:**
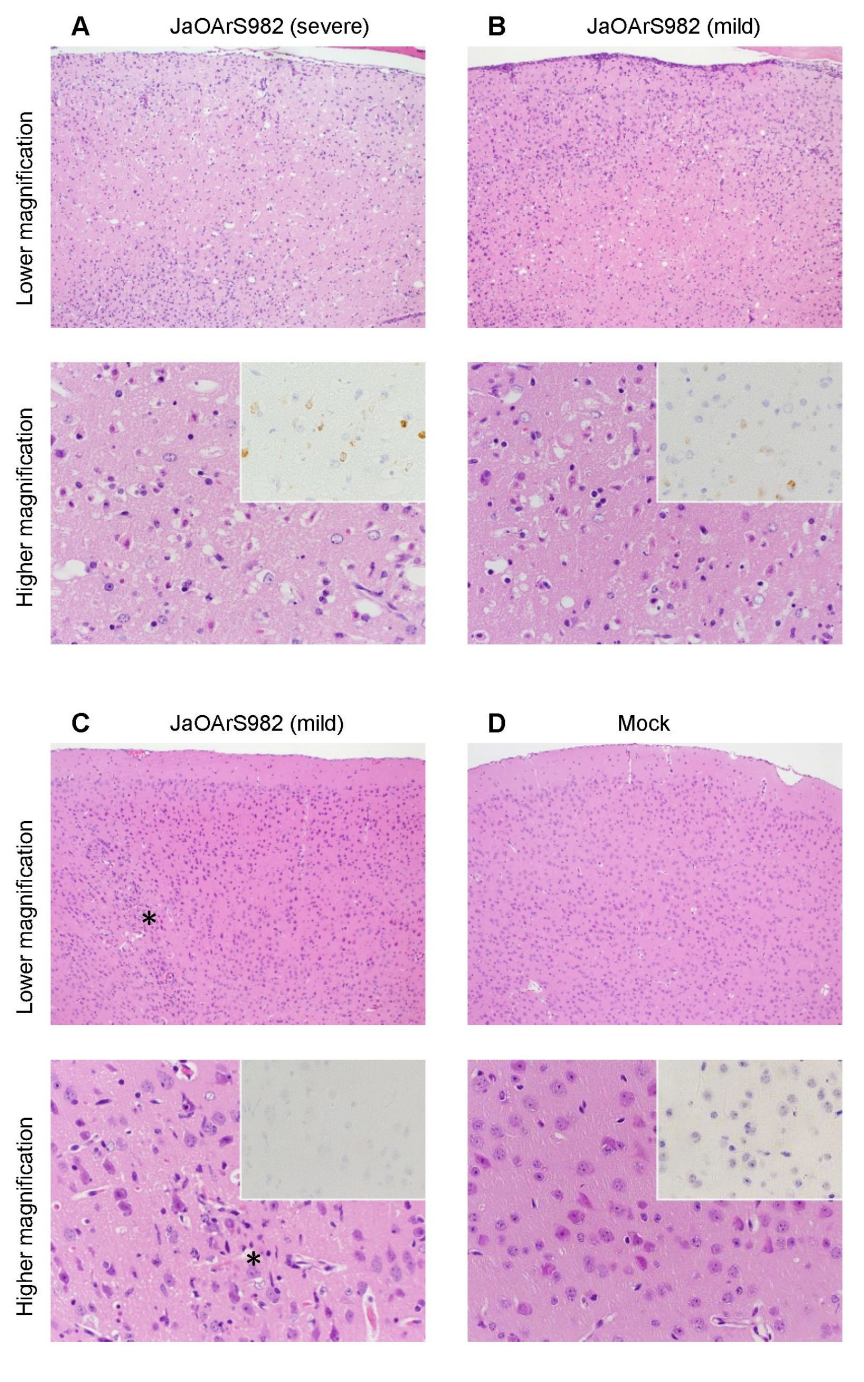
Histopathological features of brain cortex in severe and mild cases of JaOArS982-infected B6 mice. Histopathological features of B6 mice infected with 10^4^ pfu of JaOArS982 at 13 days pi. Each experiment represents five and sixteen mice of severe and mild cases, respectively. JEV antigens were detected using E-protein specific JEV antibody (insets). (A) Severe case (weight ratio: 0.63) showed acute neuronal necrosis in the brain cortex. Virus antigen-positive cells were the necrotic neurons. (B) In a mild case (weight ratio: 0.90), massive inflammatory infiltration was exhibited. Acute necrosis was seen in the area. Some JEV antigen-positive cells were detected. (C) In a mild case (weight ratio: 1.05), focal inflammatory infiltration was seen in the brain cortex (asterisk). JEV antigens were not clearly detected. (D) Mock-infected mice (weight ratio: 1.02).

Histopathological examination revealed inflammatory infiltration with mononuclear cells in the brain cortex of both severe and mild cases of JaOArS982-infected mice (Figure 4A to C). In severe cases, JEV antigens were detected in neurons, and degenerated neurons were observed in a wide area of the brain cortex and medulla (Figure 4A). On the other hand, in mild cases, there was variation of pathological features in some JaOArS982-infected mice. Other mild cases showed neuronal infections similar to those observed in severe cases but there was little neuronal degeneration in the brain cortex (Figure 4B). Other mice exhibited very limited evidence of neuronal infection and neuronal degeneration (Figure 4C). Mock-infected mice showed none of these pathological changes (Figure 4D).

In summary, both neuronal infection and CNS pathology were associated with severe disease outcome. In particular, increased levels of TNF-α and IL-10 in the brains appeared to be associated with severe disease, although it was not clear whether the increased levels were the cause or result of severe disease.

### Systemic inflammatory responses in JaOArS982-infected mice

Interestingly, the levels of TNF-α in the spleens of SG and UG mice were similar and relatively low, whereas the levels of both MHG and MLG mice were significantly higher (Figure S2A). IL-10 levels in SG, MHG and MLG mice were high compared with those in UG mice. No significant differences were observed between the SG, MHG and MLG (Figure S2A). On the other hand, the IFN-γ level in SG mice was lower than those recorded in the UG, MHG and MLG (Figure S2A). There were no significant differences of IL-2, IL-4 and IL-5 levels between UG, SG, MHG and MLG mice (Figure S2A).

Some mice in the SG showed high levels of TNF-α in the serum, although no significant difference was observed when compared with other groups (Figure S2B). IL-10 in the serum of SG mice was significantly increased compared with UG and MHG mice (Figure S2B). Corticosterone levels in the serum were also significantly increased in SG mice compared with other groups (Figure S2B). Corticosterone, a major glucocorticoid hormone, is a strong immunosuppressant and is elevated under stress response conditions such as those preceding death [55,56]. Furthermore, severe cases resulting from infection with JaOArS982 exhibited a significant reduction of CD4^+^ and CD8^+^ doubly-positive cells in the thymus (Figure S2C). Thymic depletion and body weight loss are the main features of the systemic stress response [55,56]. These observations therefore suggest that SG mice exhibited a severe systemic stress response accompanied by immune suppression. Thus, the roles of inflammatory cytokines appeared to be different in peripheral and CNS tissues.

### TNF-α and IL-10 protect mice from fatal infection with JaOArS982 virus

**Figure 5:**
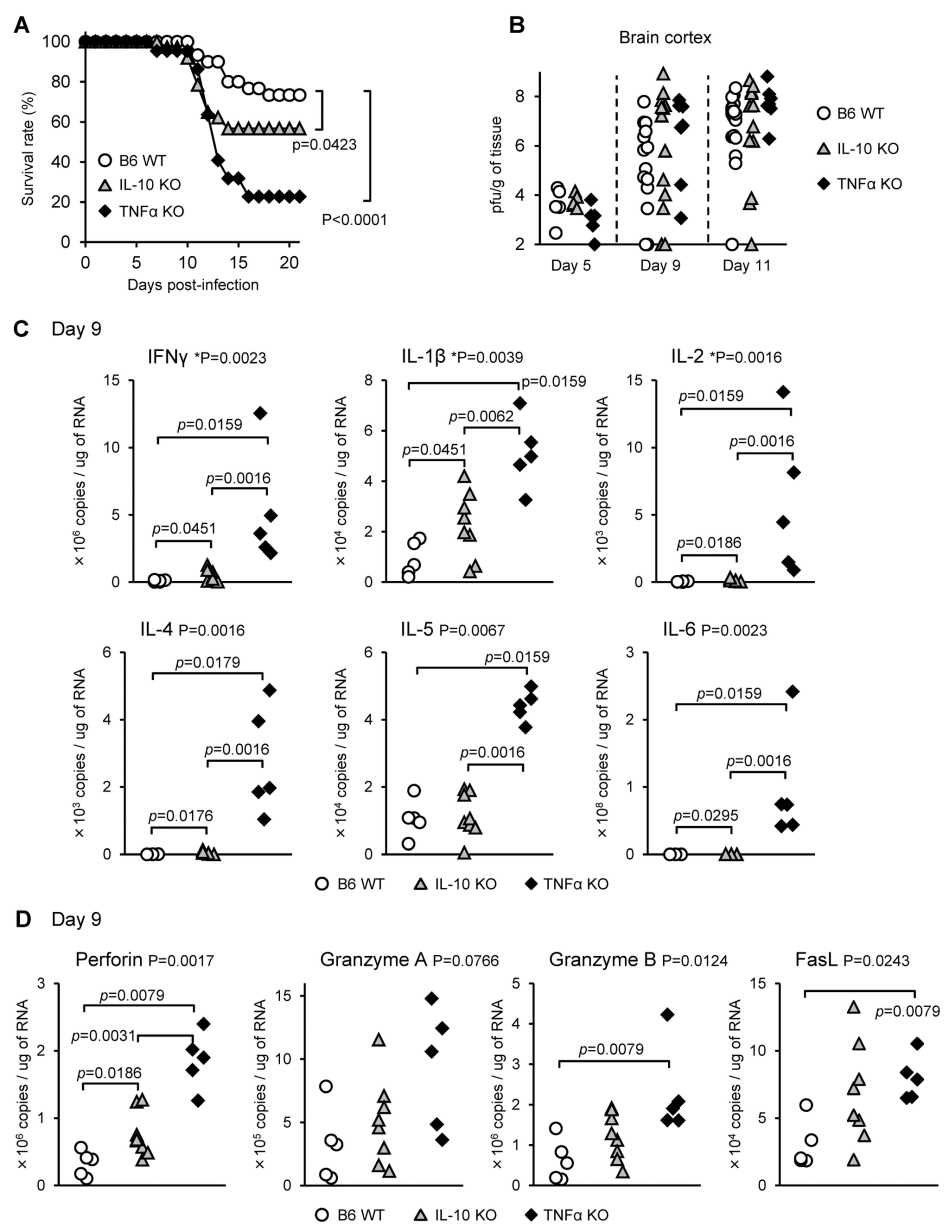
Mortality and pathogenicity of TNF-α and IL-10 KO mice infected with JaOArS982. (A) Survival curves of IL-10 KO (n=37) and TNF-α KO (n=22) B6 mice following subcutaneous infections with 10^4^ pfu of JaOArS982. WT B6 mice (n=30) show the same data as Figure 1B. P: Gehan-Breslow-Wilcoxon Test. (B) Viral loads in the brain cortex of WT (Day 5: n=5, Day 9: n=15, Day 11: n=20), IL-10 KO (Day 5: n=5, Day 9: n=15, Day 11: n=12) and TNF-α KO (Day 5: n=5, Day 9: n=7, Day 11: n=7) mice following subcutaneous infections with 10^4^ pfu of JaOArS982 at 5, 9 and 11 days pi. WT B6 mice at 5 and 9 days pi show the same dataset as Figure 1D. (C and D) mRNA levels of cytokines in the brain cortex of WT, TNF-α KO and IL-10 KO mice infected with 10^4^ pfu of JaOArS982 at 9 days pi. The levels of IFNγ, IL-1β, IL-2, IL-4, IL-5 and IL-6D (C) and perforin, granzyme A, granzyme B and FasL (D) were quantified by real-time PCR. WT B6 mice show the same data as Figure 1E and Supplemental Figure 1. P: Kruskal-Wallis test, *p*: Mann Whitney test.

To investigate in more detail, the role of TNF-α and IL-10 during JEV infection, we infected TNF-α KO and IL-10 KO B6 mice with JaOArS982, and observed the disease courses compared with those of infected fully immunocompetent B6 mice. Unexpectedly, the mortality rates of TNF-α KO and IL-10 KO mice were increased compared with those of WT mice (77.3%, 43.2% and 26.7%, respectively) (Figure 5A). MSTs of fatal cases in TNF-α KO and IL-10 KO mice (12.6 ± 1.05 and 11.5 ± 0.80 days) were significantly shorter than those of WT mice (15.5 ± 2.14 days) (p=0.0087 and p=0.0039, respectively). Consequently, these observations indicate that TNF-α and IL-10 protect significant proportions of mice from fatal infection by pathogenic JaOArS982 virus. Importantly, TNF-α had a particularly pronounced protective effect.

### Viral loads in the brains of IL-10 KO and TNF-α KO mice infected with JaOArS982

Following inoculation with JaOArS982 virus, there were no significant differences of infectious viral loads in the brain cortex between WT, IL-10 KO and TNF-α KO at 5, 9 and 11 days pi (Figure 5B). However within individual mice in each mouse group the range of viral infectivity varied from the lowest detection limit to 10^9^ pfu/g of tissue at 9 and 11 days pi (Figure 5B). It therefore appears that the increased mortality in IL-10 KO and TNF-α KO mouse was not simply due to the increased viral loads in the brains, but other factors must also have contributed to the fatal disease in these KO mice.

### Enhanced expression of proinflammatory cytokines in the brains of IL-10 KO and TNF-α KO mice infected with JaOArS982 virus

It was difficult to distinguish between dying and recovering mice on the basis of their clinical signs at 9 to 11 days pi. However, high viral loads in the brain cortex appeared to be necessary for fatal outcome. Thus, we compared the inflammatory responses in the brain cortex of mice that contained high viral loads with more than 10^6^ pfu per g of tissue (Figure 5B). Surprisingly, TNF-α KO mice exhibited significantly enhanced levels of IFN-γ, IL-1β, IL-2, IL-4, IL-5 and IL-6 in the brain when compared with the WT and IL-10 KO mice at 9 and 11 days pi (Figure 5C and Figure S3A). Furthermore, at 5 days pi, the levels of IL-4 and IL-5 were higher in TNF-α KO (Figure S3B). IL-10 KO mice also exhibited the increased levels of IFN-γ, IL-1β, IL-2, IL-4 and IL-6 compared with those of WT mice at 9 days pi, although the differences were smaller than those between TNF-α KO and WT mice (Figure 5C). Uninfected mice showed some significant differences of cytokine levels between the three groups, but the levels were very low compared with infected mice and were not significant (Figure S3C).

Furthermore, the levels of perforin, granzyme B and FasL at 9 days pi, and granzyme A at 11 days pi were significantly increased in TNF-α KO mice compared with those of WT mice (Figure 5D and Figure S3D), whereas IL-10 KO mice exhibited the increased level of perforin at 9 days pi (Figure 5D). These cytokines are associated with immune-mediated neurodegeneration.

These findings suggest that immunopathological effects in the CNS contribute to the accelerated mortality in TNF-α KO mice infected with JaOArS982. Thus, IL-10 and in particular TNF-α mediate immunomodulatory effects against such inflammatory responses.

### TNF-α KO mice display severe neuronal degeneration but no quantitative differences of infiltrated cells when compared with brains of WT mice

**Figure 6:**
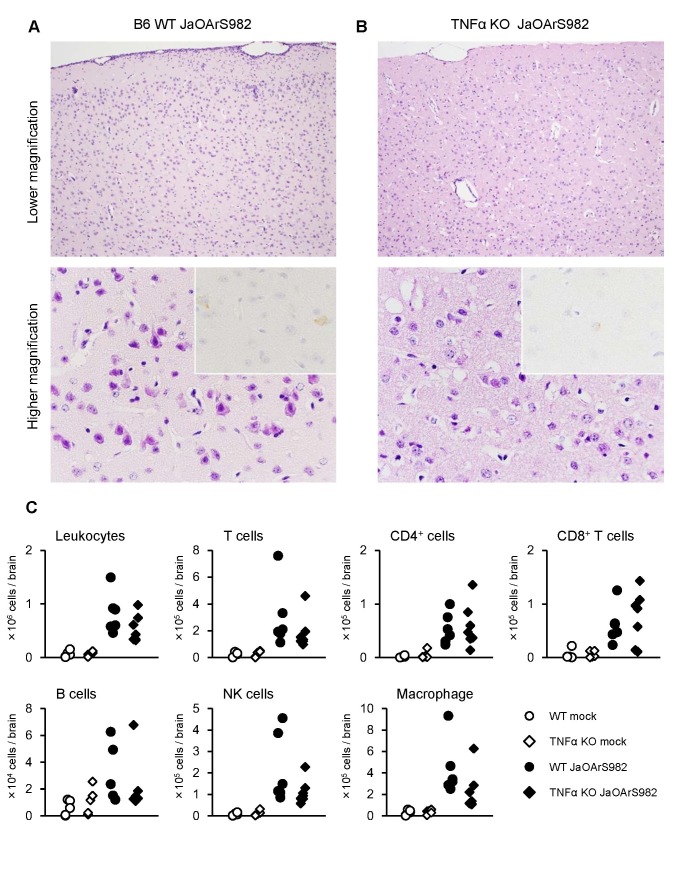
Neuronal degeneration and inflammation in the brains of TNF-α KO mice infected with JaOArS982. (A and B) Histopathological features of WT (A) and TNF-α KO (B) mice infected with 10^4^ pfu of JaOArS982 at 10 days pi. JEV antigens were detected using E-protein specific JEV antibody (insets). Each experiment represents six and five mice of WT and TNF-α KO, respectively. Inflammatory reactions and neuronal degeneration were seen in the WT mice. The TNFa KO mice showed acute necrotic changes in the brain cortex. (C) Number of infiltrating leukocytes, T cells (CD3^+^), CD4^+^ T cells, CD8^+^ T cells, B cells (CD19^+^), NK cells (NK1.1^+^) and macrophages including microglia (F4/80^+^) in brains of WT (mock: n=5, Day 10: n=6) and TNF-α KO (mock: n=5, Day 10: n=7). *p*: Mann Whitney test.

Histopathological examination of TNF-α KO mice revealed severe neuronal loss in extensive areas of brain cortex when compared with WT mice (Figure 6A and B). However, the proportion of infiltrated cells involving leukocytes (CD45), T cells (CD3, CD4 or CD8), B cells (CD19), NK cells (NK1.1) and macrophages (F4/80) did not appear to differ significantly between TNF-α KO and WT mice (Figure 6C). These observations suggest that the increased levels of cytokines in TNF-α KO mice were due to qualitative differences of their expression in inflammatory cells, rather than quantitative increases of infiltrating cytokine producing cells.

### Inflammatory responses in spleen cells of TNF-α KO mice infected with JaOArS982

In the spleens of mock-infected mice, there were no significant differences of IFN-γ levels between WT, IL-10 and TNF-α KO mice (Figure S4A). However, following JaOArS982 infection the levels of IFN-γ in TNF-α KO mice were significantly increased compared with those of WT mice at 5 and 9 days pi (Figure S4B and C). On the other hand, IL-2 and IL-4 levels in TNF-α KO mice were significantly higher than those of WT and IL-10 KO mice during mock infection (Figure S4A) and following JaOArS982 infections (Figure S4B to D). Also, the level of IL-5 in TNF-α

KO was decreased compared with WT and IL-10 KO mice at 5 days pi (Figure S4B).

These observations suggest that the patterns of cytokine levels observed in spleens were different from those of the brain and therefore that peripheral responses are unlikely to contribute to the increased disease severity in TNF-α KO mice.

### Increased levels of inflammatory cytokines in TNF-α KO mice infected with JaTH160

**Figure 7:**
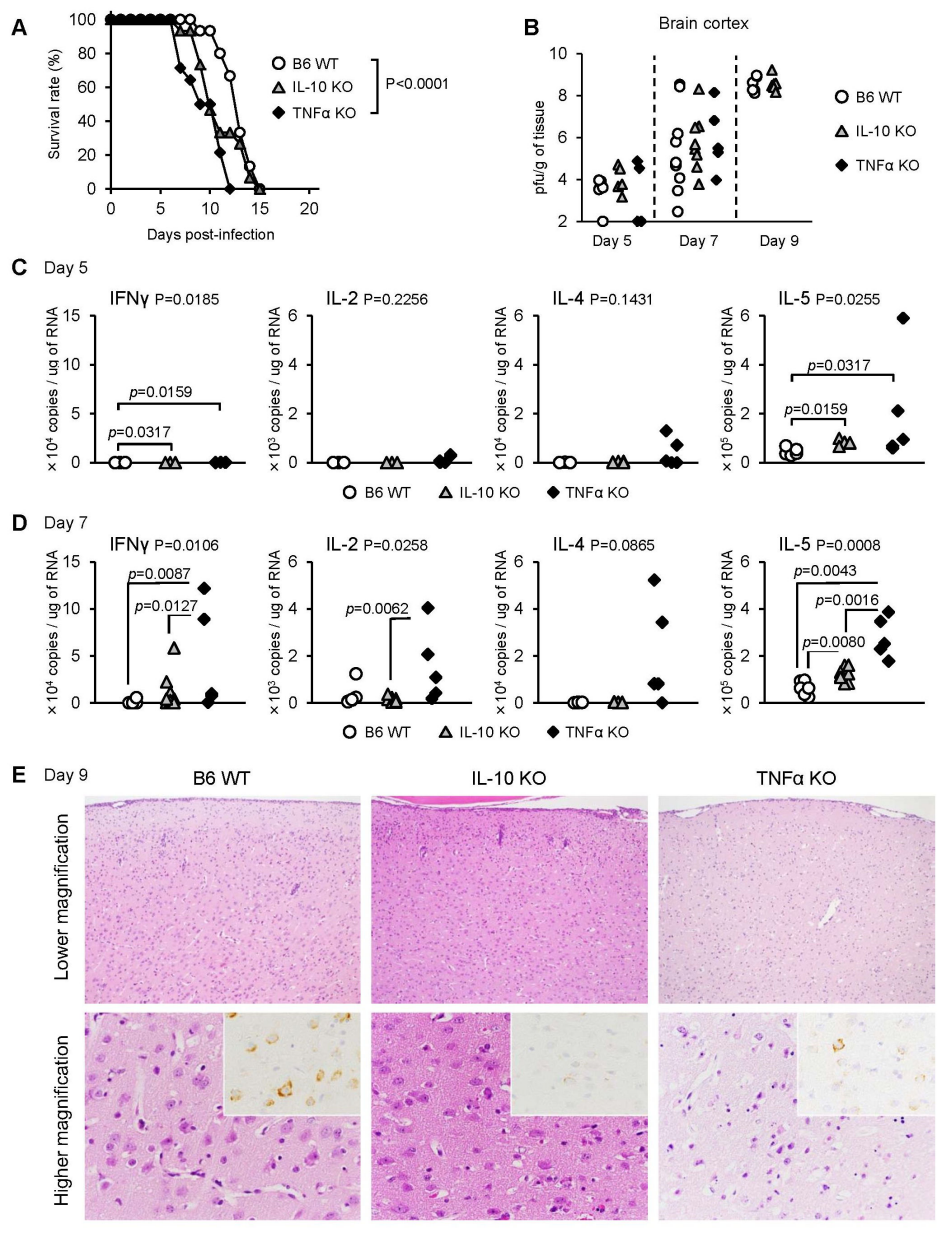
Mortality and pathogenicity of TNF-α and IL-10 KO mice infected with JaTH160. (A) Survival curves of IL-10 KO (n=15) and TNF-α KO (n=14) B6 mice following subcutaneous infections with 10^4^ pfu of JaOArS982. WT B6 mice (n=15) show the same data of Figure 1B. P: Gehan-Breslow-Wilcoxon Test. (B) Viral loads in the brain cortex of WT (Day 5: n=5, Day 7: n=9, Day 9: n=8), IL-10 KO (Day 5: n=5, Day 7: n=8, Day 9: n=6) and TNF-α KO (Day 5: n=5, Day 7: n=5) mice following subcutaneous infections with 10^4^ pfu of JaTH160 at 5, 7 and 9 days pi. Data of WT B6 mice at 5 and 9 days pi were same to Figure 1D. (C and D) mRNA levels of IFNγ, IL-2, IL-4 and IL-5 in the brain cortex of WT, TNF-α KO and IL-10 KO mice infected with 10^4^ pfu of JaTH160 at 5 (C) and 7 (D) days pi. P: Kruskal-Wallis test, *p*: Mann Whitney test. (E) Histopathological features of WT, TNF-α KO and IL-10 KO mice infected with 10^4^ pfu of JaTH160 at 9 days pi. JEV antigens were detected using E-protein specific JEV antibody (insets). Each experiment represents four, five and six mice of WT, TNF-α KO and IL-10 KO mice, respectively. The B6 WT and IL-10 KO mice showed severe inflammatory reactions in the brain cortex. On other hand, the TNF-a KO mice exhibited acute necrotic changes with slight inflammatory reactions in the brain cortex.

Although the high virulence of JaTH160 is probably attributable to viral factors, we attempted to assess whether or not TNF-α might also contribute significantly to the pathogenicity observed following infection with JaTH160 virus. Accordingly, mice were inoculated with JaTH160 virus at a challenge dose of 10^4^ pfu per mouse, all WT, IL-10 KO and TNF-α KO mouse groups died. However, TNF-α KO mice presented with significantly shorter survival times than B6 WT mice (9.57±1.19 days and 12.8±0.89 days, respectively, Mann Whitney test: p=0.0002) (Figure 7A). It is important to note that viral loads in the brains were not significantly different for either WT, TNF-α KO or IL-10 KO mice at 5 and 7 days pi (Figure 7B).

TNF-α KO mice exhibited significantly increased levels of IFN-γ and IL-5 in the brains compared with WT and/or IL-10 KO mice at 5 and 7 days pi following JaTH160 inoculation (Figure 7C and D). However, levels of IL-2 and IL-4 in the brains were not increased when WT and TNF-α KO groups at 5 and or 7 days pi were compared (Figure 7C). Moreover, levels of perforin, granzyme A, granzyme B and FasL were not increased in TNF-α KO when compared with WT mice at 7 days pi. However, histopathological data showed that TNF-α KO mice presented with severe acute necrotic changes in the brain cortex compared which was not the case for WT and IL-10 KO mice at 9 days pi (Figure 7D).

In the spleens, similar to the JaOArS982 infection, the levels of IFN-γ, IL-2 and IL-4 in IL-10 KO and TNF-α KO mice were significantly increased compared with those of WT mice at 7 days pi following JaTH160 infection, whereas the level of IL-5 was decreased in TNF-α KO (Figure S5B).

These results suggest that the shorter survival time of JaTH160-infected TNF-α KO mice when compared with WT mice may be partially attributable to an immunopathological effect, whereas direct neuronal infection is likely to be the main cause of neurodegeneration in JaTH160-infected mice.

## Discussion

This study provides the first evidence that TNF-α has an immunoregulatory effect on pro-inflammatory cytokines in the CNS during JEV infection and this results in protection from fatal disease due to infection with this virus. Following JaOArS982 virus infection, TNF-α KO mice exhibited significantly increased mortality rates when compared with WT mice. Although it has been suggested that TNF-/- mice show developmental defects of the humoral immune system including a lack of primary B cell follicles [38,57,58], TNF-α KO mice that we used in this study did not show significant depletion in the anti-JEV IgM response (data not shown) or in cytokine expression (Figure S4). In addition, no significant increases of viral propagation were observed in the peripheral and CNS tissues. Interestingly, there were no significant differences of viral load in the CNS between WT and TNF-α KO mice. However, high inflammatory responses were observed in the CNS of TNF-α KO mice. In particular, perforin, granzyme A, granzyme B and FasL, which are known to be associated with immune-mediated neurodegeneration, were significantly increased in the brains of TNF-α KO mice when compared with those of WT mice. These observations suggest that immunopathological effects contribute to the severe neuronal degeneration and fatal disease in TNF-α KO mice.

IL-10 KO mice also exhibited increased mortality and up-regulated levels of inflammatory cytokines in the CNS compared with WT mice and in common with TNF-α KO mice, there were no significant differences in viral loads. However, it is important to note that the levels of inflammatory cytokines of TNF-α KO mice and the resultant mortality were dramatically higher than those observed in IL-10 KO mice. IL-10 has an immunoregulatory function on various cells in the innate immune system including cytotoxic and helper T cells, NK cells and B cells [59]. IL-10 signaling has a negative role in immunity against WNV infection [45]. It is also known that TNF-α is a critical regulatory cytokine exerting homeostatic and pathological effects in the CSF [60]. Therefore, our data imply that TNF-α mediates greater efficacy of immunoregulatory function during JEV infection.

In preparatory studies of JEV infection, we attempted to inject TNF-α intravenously or intracerebrally after JEV inoculation to examine whether or not this improved the disease outcome. However, there was no significant improvement in the condition of the mice or protection from death. Administration of anti-mouse TNF-α antibody also showed no improvement of disease outcome. Although we cannot totally exclude the possibility that failure of TNF-α administration to improve disease outcome, may have been the result of the technical design of the experiments, different responses of TNF-α in the CNS when compared with peripheral tissues may partly explain our observations. Therefore, further investigation of the immunoregulatory mechanism of TNF-α *in vivo* and *in vitro* will be required to understand the basis of the immunopathological effects observed during JEV infection.

In this study, we focused on the variation of disease severity in mice following JaOArS982 infection to detect specific responses that may be associated with severe disease. Thus, we discriminated severe and mild cases of mice by their weight ratio at 13 days pi. Using this simple and effective approach, we had previously shown that specific immune responses were detected in severe disease cases when compared with mild cases following TBEV infection [48]. Loss of appetite probably caused the weight loss due to decreased food and water intake. However, undernourishment did not appear to be the simple cause of death, because our preliminary data showed that an infusion of glucose solution to compensate for weight loss did not prevent fatal disease.

We first considered whether or not viral quasispecies could account for the diversity of disease outcome. Our results did not support this possibility. We also identified specific immune responses including TNF-α and IL-10 up-regulation in the brains of severe cases when compared with mild cases. In human cases, an increased level of TNF-α in the CSF appears to correlate with JE disease severity [8]. Therefore, this JEV-infected mouse model appears to be a reproducible model of severe JE disease in human cases. Furthermore, from the results of increased fatality in TNF-α KO mice, we propose that increased levels of TNF-α in the brains of severe cases in WT mice were probably produced in response to the disease severity, to alleviate the pathological impact of the encephalitis.

It has been reported that immunopathological effects do contribute to flavivirus encephalitis [27]. Cytolytc leukocytes such as CD8^+^ T cells induce cytopathology during some encephalitic flavivirus infections [28–30] and these leukocytes kill virus-infected cells using two distinct mechanisms viz., Fas and granular exocytosis which involve perforin, granzyme A and B [61–64]. In TNF-α KO mice, we showed that the increased levels of inflammatory cytokines including Fas and the granular exocytosis correlated with severe encephalitis and fatal outcome. However, in WT mice, the apparent immunopathological features were not observed in dying mice 13 days pi. Although it was difficult to identify dying and surviving mice before 13 days pi by their clinical signs, JaOArS982-infected mice showed varying levels of Fas and granular exocytosis in the brains at 11 days pi and some of them exhibited similar or higher levels compared with the TNF-α KO mice (Figure S3D). Thus, fatal cases may exhibit severe encephalitis caused by immunopathological responses during the early phase of infection and thereafter severe clinical signs may appear in some mice.

JaOArS982-infected mice exhibited a variety of immune responses and different prognoses in individual mice. However, it was not clear how the immune response differentiated between dying and recovering mice. In order to explain these variable immune responses, we previously showed that specific T cell receptor (TCR) repertoires were present in dying mice during TBEV infection [65]. Furthermore, we also showed that specific TCR repertoires were detected in the dying mice compared with the recovering mice following JaOArS982 infection (Shirai, et al., unpublished results). These data raise the possibility that there may be a variety of specific T cell clones effecting either protective or pathogenetic functions in dying and recovering mice.

Dose independent mortality induced by encephalitic flaviviruses has been recognized but has been an unresolved problem since the 1940’s [27,47]. Recently, it was suggested that induction of more vigorous innate immune responses might control early virus dissemination following increasing infectious challenge doses of virus [6,26,66]. We have also recently discovered that interferon alpha receptor knockout induces dose-dependent mortality following extraneural infection with JaOArS982 (Hayasaka, et al., unpublished results). In addition, we previously reported that late death following TBEV infection appears to be a key feature of dose independent mortality within the encephalitic flaviviruses [48]. In the current study, JaOArS982-infected mice also displayed increased times to death and the variation of acquired immune responses which either showed protective or pathological effects, appeared to be correlated with severe disease. Therefore, we propose that in addition to innate immune response, subsequent acquired immune responses, which varied contingently in individuals, appeared to be a determining factor associated with dose-independent mortality.

Interestingly, JaTH160-infected mice did not show increased levels of TNF-α in the spleen at 5 and 9 days pi. However, it is uncertain if the low level of TNF-α in the spleen directly contributed to the subsequent CNS infection and the neuropathogenesis during JaTH160 infection. It is important to note that there were 17 amino acid differences in the genomic sequences of JaTH160 and JaOArS982. Therefore, it will be important to determine whether or not specific amino acid substitutions can influence TNF-α expression and thus contribute to the pathogenesis of the lethal process during JaTH160 infection.

**Figure 8:**
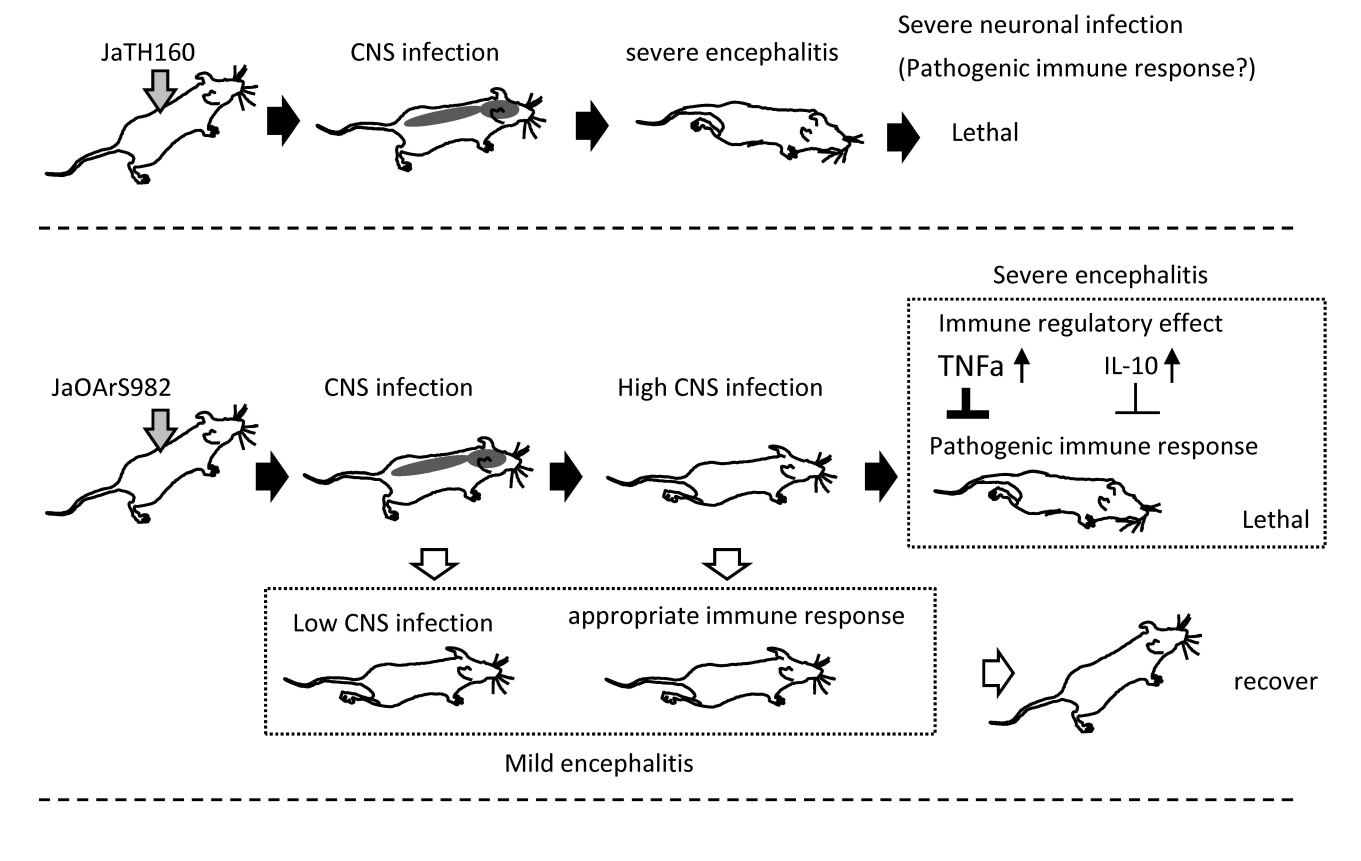
Proposed model of the mechanism of fatal disease following JEV infection in a mouse model.

In conclusion, JaTH160-infected mice developed severe encephalitis and all mice died due to severe infections of the CNS (Figure 8). On the other hand, JaOArS982-infected mice exhibited varying degrees of encephalitis and different prognoses (Figure 8). We therefore propose that fatal outcome is attributable both to immunopathological changes in addition to high levels of CNS infection. At this stage we cannot define the critical factors involved in the immunopathogenetic process (Figure 8). Furthermore, up-regulation of TNF-α and IL-10 in the brain appear to be important determinants of the pathogenetic response (Figure 8). Clearly, further elucidation of the contribution of immunopathology and the suppressive impact of TNF-α, are important priorities to enable the development of effective treatment strategies for JE.

## Supporting Information

Figure S1 Cytokine levels of spleen in B6 mice infected with JaOArS982 and JaTH160. (A) mRNA levels of IL-4 and IL-5 quantified by real-time PCR in the brain cortex of B6 mice infected with 10^4^ pfu of JaOArS982 (Day 5: n=5, Day 9: n=12), JaTH160 (Day 5: n=5, Day 9: n=5) and mock (n=8). P: Mann Whitney test. (B) mRNA levels of TNF-α, IFNγ, IL-2, IL-10, IL-4 and IL-5 quantified by real-time PCR in the spleen of B6 mice infected with 10^4^ pfu of JaOArS982 (Day 5: n=5, Day 9: n=12), JaTH160 (Day 5: n=5, Day 9: n=5) and mock (n=8). P: Mann Whitney test.
(TIF)

Figure S2 Cytokine levels of spleen in severe and mild cases of JaOArS982-infected mice. (A) mRNA levels of TNF-α, IL-10, IFNγ, IL-2, IL-4 and IL-5 quantified by real-time PCR in the brain cortex of JaOArS982-infected B6 mice at 13 days pi. Uninfected group: U (n=8), Severe group: S (n=8), Mild group with high viral load of >10^6^ pfu/g of brain tissue: MH (n=11), Mild group with low viral load of <10^6^ pfu/g of brain tissue: ML (n=13). P: Kruskal-Wallis test, *p*: Mann Whitney test. (B) The levels of IL-10, TNF-α and corticosterone measured by enzyme-linked immunosorbent assay in the plasma of JaOArS982-infected B6 mice at 13 days pi Uninfected group (U group, n=6), Severe group (S group, n=6), Mild group with high viral load of >10^6^ pfu/g of brain tissue (MH group, n=7), Mild group with low viral load of <10^6^ pfu/g of brain tissue (ML group, n=6). P: Kruskal-Wallis test, *p*: Mann Whitney test. (C) CD4 and CD8 expressions of thymocytes from mock, mild and severe cases of JaOArS982-infected B6 mice at 13 days pi. Each experiment represents four and fifteen mice of severe and mild cases, respectively.
(TIF)

Figure S3 Cytokine levels of brains in TNF-α and IL-10 KO mice infected with JaOArS982. (A to C) mRNA levels of IFNγ, IL-2, IL-4, IL-5 quantified by real-time PCR in the brain cortex of WT, TNF-α and IL-10 mice infected with 10^4^ pfu of JaOArS982 at 11 (A) and 5 (B) days pi and uninfected mice (C). (D and E) mRNA levels of perforin, granzyme A, granzyme B and FasL in the brain cortex of WT, TNF-α and IL-10 mice infected JaOArS982 at 11 days pi (D) and uninfected mice (E).
(TIF)

Figure S4 Cytokine levels of spleens in TNF-α and IL-10 KO mice infected with JaOArS982. (A) mRNA levels of IFNγ, IL-2, IL-4, IL-5 quantified by real-time PCR in the spleen of WT, TNF-α and IL-10 mice infected with mock (A) and 10^4^ pfu of JaOArS982 at 5 (B), 9 (C) and 11 (D) days pi. P: Kruskal-Wallis test, *p*: Mann Whitney test.
(TIF)

Figure S5 Cytokine levels of brain and spleen in TNF-α and IL-10 KO mice infected with JaTH160. (A) mRNA levels of perforin, granzyme A, granzyme B and FasL in the brain cortex of WT, IL-10 and TNF-α mice at 7 days pi. P: Kruskal-Wallis test. (B) mRNA levels of IFNγ, IL-2, IL-4, IL-5 in the spleen of WT, IL-10 and TNF-α mice at 5 days pi. P: Kruskal-Wallis test, *p*: Mann Whitney test.
(TIF)

## References

[1] Gubler JD, Kuno G, Markoff L (2007) Flaviviruses. In: Knipe DM Howley PM Griffin DE Lamb RA Straus SE Fields virology. Philadelphia, PA: Lippincott Williams & Wilkins, a. Wolters Kluwer Business pp. 1153-1252.

[2] Erlanger TE, Weiss S, Keiser J, Utzinger J, Wiedenmayer K (2009) Past, present, and future of Japanese encephalitis. Emerg Infect Dis 15: 1-7. doi:10.3201/eid1506.081196. PubMed: 19116041. 19116041 10.3201/eid1501.080311 PMC2660690

[3] Konishi E, Kitai Y, Tabei Y, Nishimura K, Harada S (2010) Natural Japanese encephalitis virus infection among humans in west and east Japan shows the need to continue a vaccination program. Vaccine 28: 2664-2670. doi:10.1016/j.vaccine.2010.01.008. PubMed: 20080072. 20080072 10.1016/j.vaccine.2010.01.008

[4] Misra UK, Kalita J (2010) Overview: Japanese encephalitis. Prog Neurobiol 91: 108-120. doi:10.1016/j.pneurobio.2010.01.008. PubMed: 20132860. 20132860 10.1016/j.pneurobio.2010.01.008

[5] Tsai TF (2000) New initiatives for the control of Japanese encephalitis by vaccination: minutes of a WHO/CVI meeting, Bangkok, Thailand, 13-15 October 1998. Vaccine 18 Suppl 2: 1-25. doi:10.1016/S0264-410X(99)00449-1. PubMed: 10821969. 10821969 10.1016/s0264-410x(00)00037-2

[6] Larena M, Lobigs M (2011) Immunobiology of Japanese Encephalitis Virus. In: Ruzek D FLAVIVIRUS ENCEPHALITIS. Croatia. Intech: 317-338.

[7] Winter PM, Dung NM, Loan HT, Kneen R, Wills B et al. (2004) Proinflammatory cytokines and chemokines in humans with Japanese encephalitis. J Infect Dis 190: 1618-1626. doi:10.1086/423328. PubMed: 15478067. 15478067 10.1086/423328

[8] Ravi V, Parida S, Desai A, Chandramuki A, Gourie-Devi M et al. (1997) Correlation of tumor necrosis factor levels in the serum and cerebrospinal fluid with clinical outcome in Japanese encephalitis patients. J Med Virol 51: 132-136. doi:10.1002/(SICI)1096-9071(199702)51:2. PubMed: 9021544. 9021544

[9] Kimura T, Sasaki M, Okumura M, Kim E, Sawa H (2010) Flavivirus encephalitis: pathological aspects of mouse and other animal models. Vet Pathol 47: 806-818. doi:10.1177/0300985810372507. PubMed: 20551474. 20551474 10.1177/0300985810372507

[10] German AC, Myint KS, Mai NT, Pomeroy I, Phu NH et al. (2006) A preliminary neuropathological study of Japanese encephalitis in humans and a mouse model. Trans R Soc Trop Med Hyg 100: 1135-1145. doi:10.1016/j.trstmh.2006.02.008. PubMed: 16814333. 16814333 10.1016/j.trstmh.2006.02.008

[11] Hase T, Dubois DR, Summers PL (1990) Comparative study of mouse brains infected with Japanese encephalitis virus by intracerebral or intraperitoneal inoculation. Int J Exp Pathol 71: 857-869. PubMed: 2177623. 2177623 PMC2002376

[12] Garcia-Tapia D, Hassett DE, Mitchell WJ Jr., Johnson GC, Kleiboeker SB (2007) West Nile virus encephalitis: sequential histopathological and immunological events in a murine model of infection. J Neurovirol 13: 130-138. doi:10.1080/13550280601187185. PubMed: 17505981. 17505981 10.1080/13550280601187185

[13] Albrecht P (1968) Pathogenesis of neurotropic arbovirus infections. Curr Top Microbiol Immunol 43: 44-91. doi:10.1007/978-3-642-46118-7_2. PubMed: 4970327. 4970327 10.1007/978-3-642-46118-7_2

[14] Burke SD, Monath PT (2001) Flaviviruses. In: Knipe DM Howley PM Griffin DE Lamb RA Martin MA Fields virology. Philadelphia, PA: Lippincott Williams & Wilkins pp. 991-1041.

[15] Byrne SN, Halliday GM, Johnston LJ, King NJ (2001) Interleukin-1beta but not tumor necrosis factor is involved in West Nile virus-induced Langerhans cell migration from the skin in C57BL/6 mice. J Invest Dermatol 117: 702-709. doi:10.1046/j.0022-202x.2001.01454.x. PubMed: 11564180. 11564180 10.1046/j.0022-202x.2001.01454.x

[16] Dumpis U, Crook D, Oksi J (1999) Tick-borne encephalitis. Clin Infect Dis 28: 882-890. doi:10.1086/515195. PubMed: 10825054. 10825054 10.1086/515195

[17] Robertson SJ, Mitzel DN, Taylor RT, Best SM, Bloom ME (2009) Tick-borne flaviviruses: dissecting host immune responses and virus countermeasures. Immunol Res 43: 172-186. doi:10.1007/s12026-008-8065-6. PubMed: 18841330. 18841330 10.1007/s12026-008-8065-6 PMC2774773

[18] Samuel MA, Diamond MS (2006) Pathogenesis of West Nile Virus infection: a balance between virulence, innate and adaptive immunity, and viral evasion. J Virol 80: 9349-9360. doi:10.1128/JVI.01122-06. PubMed: 16973541. 16973541 10.1128/JVI.01122-06 PMC1617273

[19] Turtle L, Griffiths MJ, Solomon T (2012) Encephalitis caused by flaviviruses. QJM 105: 219-223. doi:10.1093/qjmed/hcs013. PubMed: 22367423. 22367423 10.1093/qjmed/hcs013 PMC3285924

[20] Klein RS, Lin E, Zhang B, Luster AD, Tollett J et al. (2005) Neuronal CXCL10 directs CD8+ T-cell recruitment and control of West Nile virus encephalitis. J Virol 79: 11457-11466. doi:10.1128/JVI.79.17.11457-11466.2005. PubMed: 16103196. 16103196 10.1128/JVI.79.17.11457-11466.2005 PMC1193600

[21] Pinto AK, Daffis S, Brien JD, Gainey MD, Yokoyama WM et al. (2011) A temporal role of type I interferon signaling in CD8+ T cell maturation during acute West Nile virus infection. PLOS Pathog 7: e1002407 PubMed: 22144897. 22144897 10.1371/journal.ppat.1002407 PMC3228803

[22] Shrestha B, Diamond MS (2004) Role of CD8+ T cells in control of West Nile virus infection. J Virol 78: 8312-8321. doi:10.1128/JVI.78.15.8312-8321.2004. PubMed: 15254203. 15254203 10.1128/JVI.78.15.8312-8321.2004 PMC446114

[23] Shrestha B, Pinto AK, Green S, Bosch I, Diamond MS (2012) CD8+ T cells use TRAIL to restrict West Nile virus pathogenesis by controlling infection in neurons. J Virol 86: 8937-8948. doi:10.1128/JVI.00673-12. PubMed: 22740407. 22740407 10.1128/JVI.00673-12 PMC3416144

[24] Sitati E, McCandless EE, Klein RS, Diamond MS (2007) CD40-CD40 ligand interactions promote trafficking of CD8+ T cells into the brain and protection against West Nile virus encephalitis. J Virol 81: 9801-9811. doi:10.1128/JVI.00941-07. PubMed: 17626103. 17626103 10.1128/JVI.00941-07 PMC2045405

[25] Zhang B, Chan YK, Lu B, Diamond MS, Klein RS (2008) CXCR3 mediates region-specific antiviral T cell trafficking within the central nervous system during West Nile virus encephalitis. J Immunol 180: 2641-2649. PubMed: 18250476. 18250476 10.4049/jimmunol.180.4.2641

[26] Larena M, Regner M, Lee E, Lobigs M (2011) Pivotal role of antibody and subsidiary contribution of CD8+ T cells to recovery from infection in a murine model of Japanese encephalitis. J Virol 85: 5446-5455. doi:10.1128/JVI.02611-10. PubMed: 21450826. 21450826 10.1128/JVI.02611-10 PMC3094953

[27] King NJ, Getts DR, Getts MT, Rana S, Shrestha B et al. (2007) Immunopathology of flavivirus infections. Immunol Cell Biol 85: 33-42. doi:10.1038/sj.icb.7100012. PubMed: 17146465. 17146465 10.1038/sj.icb.7100012

[28] Růzek D, Salát J, Palus M, Gritsun TS, Gould EA et al. (2009) CD8+ T-cells mediate immunopathology in tick-borne encephalitis. Virology 384: 1-6. doi:10.1016/j.virol.2008.11.023. PubMed: 19070884. 19070884 10.1016/j.virol.2008.11.023

[29] Wang Y, Lobigs M, Lee E, Müllbacher A (2003) CD8+ T cells mediate recovery and immunopathology in West Nile virus encephalitis. J Virol 77: 13323-13334. doi:10.1128/JVI.77.24.13323-13334.2003. PubMed: 14645588. 14645588 10.1128/JVI.77.24.13323-13334.2003 PMC296062

[30] Licon Luna RM, Lee E, Müllbacher A, Blanden RV, Langman R et al. (2002) Lack of both Fas ligand and perforin protects from flavivirus-mediated encephalitis in mice. J Virol 76: 3202-3211. doi:10.1128/JVI.76.7.3202-3211.2002. PubMed: 11884544. 11884544 10.1128/JVI.76.7.3202-3211.2002 PMC136025

[31] Smith JA, Das A, Ray SK, Banik NL (2012) Role of pro-inflammatory cytokines released from microglia in neurodegenerative diseases. Brain. Res Bull 87: 10-20. doi:10.1016/j.brainresbull.2011.10.004. 10.1016/j.brainresbull.2011.10.004 PMC9827422 22024597

[32] Ghoshal A, Das S, Ghosh S, Mishra MK, Sharma V et al. (2007) Proinflammatory mediators released by activated microglia induces neuronal death in Japanese encephalitis. Glia 55: 483-496. doi:10.1002/glia.20474. PubMed: 17203475. 17203475 10.1002/glia.20474

[33] Thongtan T, Thepparit C, Smith DR (2012) The involvement of microglial cells in Japanese encephalitis infections. Clin Dev Immunol, 2012: 2012: 890586. PubMed: 22919405 10.1155/2012/890586 PMC3420229 22919405

[34] Swarup V, Ghosh J, Das S, Basu A (2008) Tumor necrosis factor receptor-associated death domain mediated neuronal death contributes to the glial activation and subsequent neuroinflammation in Japanese encephalitis. Neurochem Int 52: 1310-1321. doi:10.1016/j.neuint.2008.01.014. PubMed: 18325634. 18325634 10.1016/j.neuint.2008.01.014

[35] Swarup V, Das S, Ghosh S, Basu A (2007) Tumor necrosis factor receptor-1-induced neuronal death by TRADD contributes to the pathogenesis of Japanese encephalitis. J Neurochem 103: 771-783. doi:10.1111/j.1471-4159.2007.04790.x. PubMed: 17666051. 17666051 10.1111/j.1471-4159.2007.04790.x

[36] Chen CJ, Ou YC, Chang CY, Pan HC, Liao SL et al. (2011) TNF-alpha and IL-1beta mediate Japanese encephalitis virus-induced RANTES gene expression in astrocytes. Neurochem Int 58: 234-242. doi:10.1016/j.neuint.2010.12.009. PubMed: 21167894. 21167894 10.1016/j.neuint.2010.12.009

[37] Chen CJ, Ou YC, Chang CY, Pan HC, Liao SL et al. (2012) Glutamate released by Japanese encephalitis virus-infected microglia involves TNF-alpha signaling and contributes to neuronal death. Glia 60: 487-501. doi:10.1002/glia.22282. PubMed: 22144112. 22144112 10.1002/glia.22282

[38] Shrestha B, Zhang B, Purtha WE, Klein RS, Diamond MS (2008) Tumor necrosis factor alpha protects against lethal West Nile virus infection by promoting trafficking of mononuclear leukocytes into the central nervous system. J Virol 82: 8956-8964. doi:10.1128/JVI.01118-08. PubMed: 18632856. 18632856 10.1128/JVI.01118-08 PMC2546880

[39] Szretter KJ, Samuel MA, Gilfillan S, Fuchs A, Colonna M et al. (2009) The immune adaptor molecule SARM modulates tumor necrosis factor alpha production and microglia activation in the brainstem and restricts West Nile Virus pathogenesis. J Virol 83: 9329-9338. doi:10.1128/JVI.00836-09. PubMed: 19587044. 19587044 10.1128/JVI.00836-09 PMC2738257

[40] Zhang B, Patel J, Croyle M, Diamond MS, Klein RS (2010) TNF-alpha-dependent regulation of CXCR3 expression modulates neuronal survival during West Nile virus encephalitis. J Neuroimmunol 224: 28-38. doi:10.1016/j.jneuroim.2010.05.003. PubMed: 20579746. 20579746 10.1016/j.jneuroim.2010.05.003 PMC2910216

[41] Pestka S, Krause CD, Sarkar D, Walter MR, Shi Y et al. (2004) Interleukin-10 and related cytokines and receptors. Annu Rev Immunol 22: 929-979. doi:10.1146/annurev.immunol.22.012703.104622. PubMed: 15032600. 15032600 10.1146/annurev.immunol.22.012703.104622

[42] Biswas SM, Kar S, Singh R, Chakraborty D, Vipat V et al. (2010) Immunomodulatory cytokines determine the outcome of Japanese encephalitis virus infection in mice. J Med Virol 82: 304-310. doi:10.1002/jmv.21688. PubMed: 20029807. 20029807 10.1002/jmv.21688

[43] Saxena V, Mathur A, Krishnani N, Dhole TN (2008) An insufficient anti-inflammatory cytokine response in mouse brain is associated with increased tissue pathology and viral load during Japanese encephalitis virus infection. Arch Virol 153: 283-292. doi:10.1007/s00705-007-1098-7. PubMed: 18074098. 18074098 10.1007/s00705-007-1098-7

[44] Saxena V, Mathur A, Krishnani N, Dhole TN (2008) Kinetics of cytokine profile during intraperitoneal inoculation of Japanese encephalitis virus in BALB/c mice model. Microbes Infect 10: 1210-1217. doi:10.1016/j.micinf.2008.06.015. PubMed: 18691668. 18691668 10.1016/j.micinf.2008.06.015

[45] Bai F, Town T, Qian F, Wang P, Kamanaka M et al. (2009) IL-10 signaling blockade controls murine West Nile virus infection. PLOS Pathog 5: e1000610 PubMed: 19816558. 19816558 10.1371/journal.ppat.1000610 PMC2749443

[46] Mandl CW (2005) Steps of the tick-borne encephalitis virus replication cycle that affect neuropathogenesis. Virus Res 111: 161-174. doi:10.1016/j.virusres.2005.04.007. PubMed: 15871909. 15871909 10.1016/j.virusres.2005.04.007

[47] Lennette EH (1944) Influence of age on the susceptibility of mice to infection with certain neurotropic viruses. J Immunol 49: 175-191.

[48] Hayasaka D, Nagata N, Fujii Y, Hasegawa H, Sata T et al. (2009) Mortality following peripheral infection with tick-borne encephalitis virus results from a combination of central nervous system pathology, systemic inflammatory and stress responses. Virology 390: 139-150. doi:10.1016/j.virol.2009.04.026. PubMed: 19467556. 19467556 10.1016/j.virol.2009.04.026

[49] Hayasaka D, Ivanov L, Leonova GN, Goto A, Yoshii K et al. (2001) Distribution and characterization of tick-borne encephalitis viruses from Siberia and far-eastern Asia. J Gen Virol 82: 1319-1328. PubMed: 11369875. 11369875 10.1099/0022-1317-82-6-1319

[50] Kühn R, Löhler J, Rennick D, Rajewsky K, Müller W (1993) Interleukin-10-deficient mice develop chronic enterocolitis. Cell 75: 263-274. doi:10.1016/0092-8674(93)80068-P. PubMed: 8402911. 8402911 10.1016/0092-8674(93)80068-p

[51] Taniguchi T, Takata M, Ikeda A, Momotani E, Sekikawa K (1997) Failure of germinal center formation and impairment of response to endotoxin in tumor necrosis factor alpha-deficient mice. Lab Invest 77: 647-658. PubMed: 9426403. 9426403

[52] Fujii Y, Kitaura K, Nakamichi K, Takasaki T, Suzuki R et al. (2008) Accumulation of T-cells with selected T-cell receptors in the brains of Japanese encephalitis virus-infected mice. Jpn J Infect Dis 61: 40-48. PubMed: 18219133. 18219133

[53] Nagata N, Iwata N, Hasegawa H, Fukushi S, Yokoyama M et al. (2007) Participation of both host and virus factors in induction of severe acute respiratory syndrome (SARS) in F344 rats infected with SARS coronavirus. J Virol 81: 1848-1857. doi:10.1128/JVI.01967-06. PubMed: 17151094. 17151094 10.1128/JVI.01967-06 PMC1797583

[54] Hayasaka D, Ennis FA, Terajima M (2007) Pathogeneses of respiratory infections with virulent and attenuated vaccinia viruses. Virol J 4: 22. doi:10.1186/1743-422X-4-22. PubMed: 17326843. 17326843 10.1186/1743-422X-4-22 PMC1810241

[55] Dunn AJ, Powell ML, Meitin C, Small PA Jr. (1989) Virus infection as a stressor: influenza virus elevates plasma concentrations of corticosterone, and brain concentrations of MHPG and tryptophan. Physiol Behav 45: 591-594. doi:10.1016/0031-9384(89)90078-4. PubMed: 2756050. 2756050 10.1016/0031-9384(89)90078-4

[56] Savino W (2006) The thymus is a common target organ in infectious diseases. PLOS Pathog 2: e62. doi:10.1371/journal.ppat.0020062. PubMed: 16846255. 16846255 10.1371/journal.ppat.0020062 PMC1483230

[57] Pasparakis M, Alexopoulou L, Episkopou V, Kollias G (1996) Immune and inflammatory responses in TNF alpha-deficient mice: a critical requirement for TNF alpha in the formation of primary B cell follicles, follicular dendritic cell networks and germinal centers, and in the maturation of the humoral immune response. J Exp Med 184: 1397-1411. doi:10.1084/jem.184.4.1397. PubMed: 8879212. 8879212 10.1084/jem.184.4.1397 PMC2192824

[58] Pasparakis M, Alexopoulou L, Grell M, Pfizenmaier K, Bluethmann H et al. (1997) Peyer’s patch organogenesis is intact yet formation of B lymphocyte follicles is defective in peripheral lymphoid organs of mice deficient for tumor necrosis factor and its 55-kDa receptor. Proc Natl Acad Sci U S A 94: 6319-6323. doi:10.1073/pnas.94.12.6319. PubMed: 9177215. 9177215 10.1073/pnas.94.12.6319 PMC21047

[59] Moore KW, de Waal Malefyt R, Coffman RL, O’Garra A (2001) Interleukin-10 and the interleukin-10 receptor. Annu Rev Immunol 19: 683-765. doi:10.1146/annurev.immunol.19.1.683. PubMed: 11244051. 11244051 10.1146/annurev.immunol.19.1.683

[60] Montgomery SL, Bowers WJ (2012) Tumor necrosis factor-alpha and the roles it plays in homeostatic and degenerative processes within the central nervous system. J Neuroimmune Pharmacol 7: 42-59. doi:10.1007/s11481-011-9287-2. PubMed: 21728035. 21728035 10.1007/s11481-011-9287-2

[61] Kägi D, Vignaux F, Ledermann B, Bürki K, Depraetere V et al. (1994) Fas and perforin pathways as major mechanisms of T cell-mediated cytotoxicity. Science 265: 528-530. doi:10.1126/science.7518614. PubMed: 7518614. 7518614 10.1126/science.7518614

[62] Kojima H, Shinohara N, Hanaoka S, Someya-Shirota Y, Takagaki Y et al. (1994) Two distinct pathways of specific killing revealed by perforin mutant cytotoxic T lymphocytes. Immunity 1: 357-364. doi:10.1016/1074-7613(94)90066-3. PubMed: 7533644. 7533644 10.1016/1074-7613(94)90066-3

[63] Kägi D, Ledermann B, Bürki K, Zinkernagel RM, Hengartner H (1996) Molecular mechanisms of lymphocyte-mediated cytotoxicity and their role in immunological protection and pathogenesis in vivo. Annu Rev Immunol 14: 207-232. doi:10.1146/annurev.immunol.14.1.207. PubMed: 8717513. 8717513 10.1146/annurev.immunol.14.1.207

[64] Müllbacher A, Waring P, Tha Hla R, Tran T, Chin S et al. (1999) Granzymes are the essential downstream effector molecules for the control of primary virus infections by cytolytic leukocytes. Proc Natl Acad Sci U S A 96: 13950-13955. doi:10.1073/pnas.96.24.13950. PubMed: 10570179. 10570179 10.1073/pnas.96.24.13950 PMC24171

[65] Fujii Y, Hayasaka D, Kitaura K, Takasaki T, Suzuki R et al. (2011) T-cell clones expressing different T-cell receptors accumulate in the brains of dying and surviving mice after peripheral infection with far eastern strain of tick-borne encephalitis virus. Viral Immunol 24: 291-302. doi:10.1089/vim.2011.0017. PubMed: 21830901. 21830901 10.1089/vim.2011.0017

[66] Monath TP, Guirakhoo F, Nichols R, Yoksan S, Schrader R et al. (2003) Chimeric live, attenuated vaccine against Japanese encephalitis (ChimeriVax-JE): phase 2 clinical trials for safety and immunogenicity, effect of vaccine dose and schedule, and memory response to challenge with inactivated Japanese encephalitis antigen. J Infect Dis 188: 1213-1230. doi:10.1086/378356. PubMed: 14551893. 14551893 10.1086/378356

