# Supplementary figures and images for: TNF-α Acts as an Immunoregulator in the Mouse Brain by Reducing the Incidence of Severe Disease Following Japanese Encephalitis Virus Infection
Source: PLoS One. 2013 Aug 5;8(8):e71643. doi: 10.1371/journal.pone.0071643 (PMC3733918; doi:10.1371/journal.pone.0071643)

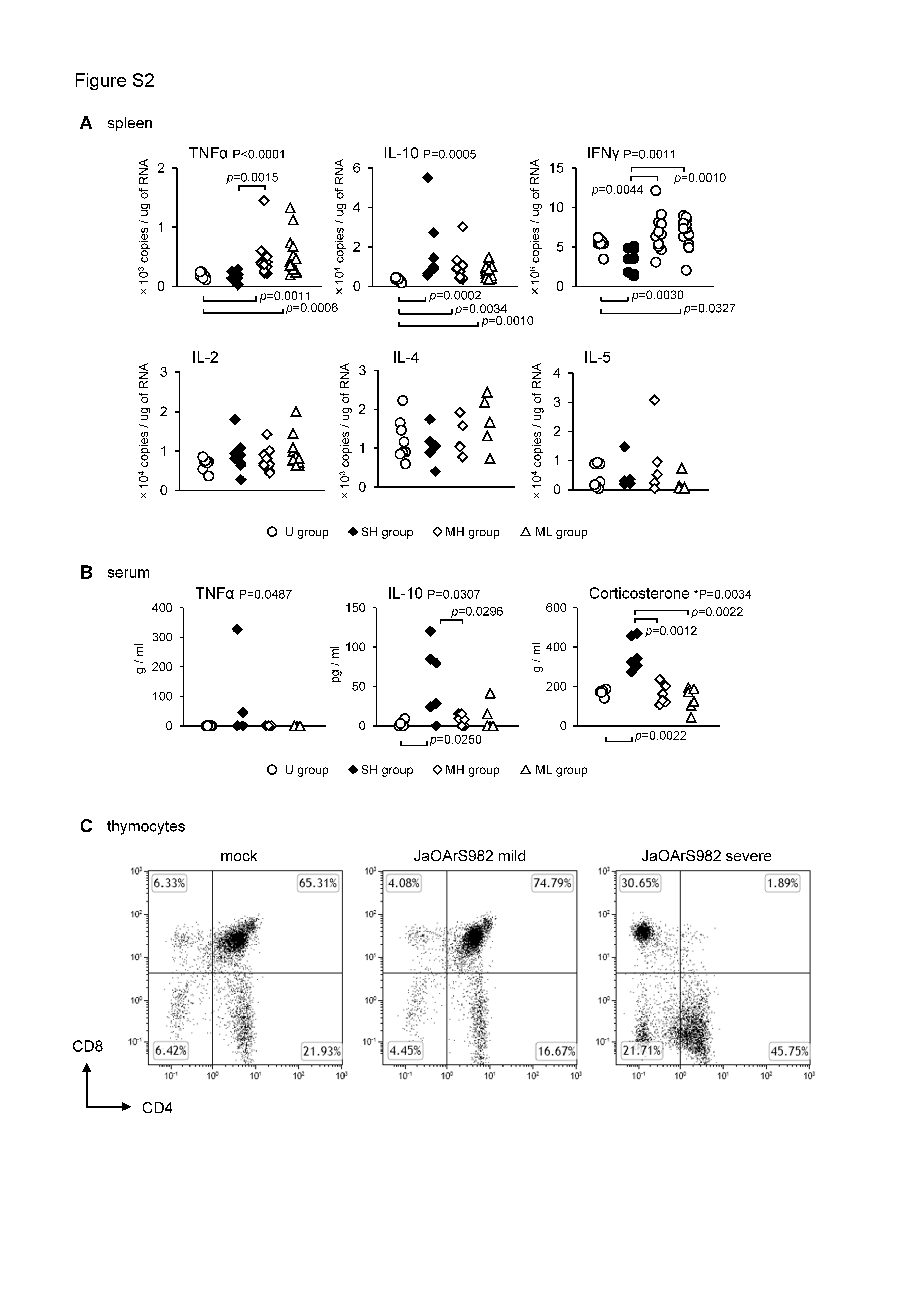

Supplement: Figure S2 — (A) mRNA levels of TNF-α, IL-10, IFNγ, IL-2, IL-4 and IL-5 quantified by real-time PCR in the brain cortex of JaOArS982-infected B6 mice at 13 days pi. Uninfected group: U (n=8), Severe group: S (n=8), Mild group with high viral load of >106 pfu/g of brain tissue: MH (n=11), Mild group with low viral load of <106 pfu/g of brain tissue: ML (n=13). P: Kruskal-Wallis test, p: Mann Whitney test. (B) The levels of IL-10, TNF-α and corticosterone measured by enzyme-linked immunosorbent assay in the plasma of JaOArS982-infected B6 mice at 13 days pi Uninfected group (U group, n=6), Severe group (S group, n=6), Mild group with high viral load of >106 pfu/g of brain tissue (MH group, n=7), Mild group with low viral load of <106 pfu/g of brain tissue (ML group, n=6). P: Kruskal-Wallis test, p: Mann Whitney test. (C) CD4 and CD8 expressions of thymocytes from mock, mild and severe cases of JaOArS982-infected B6 mice at 13 days pi. Each experiment represents four and fifteen mice of severe and mild cases, respectively. (TIF) [file pone.0071643.s002.tif]

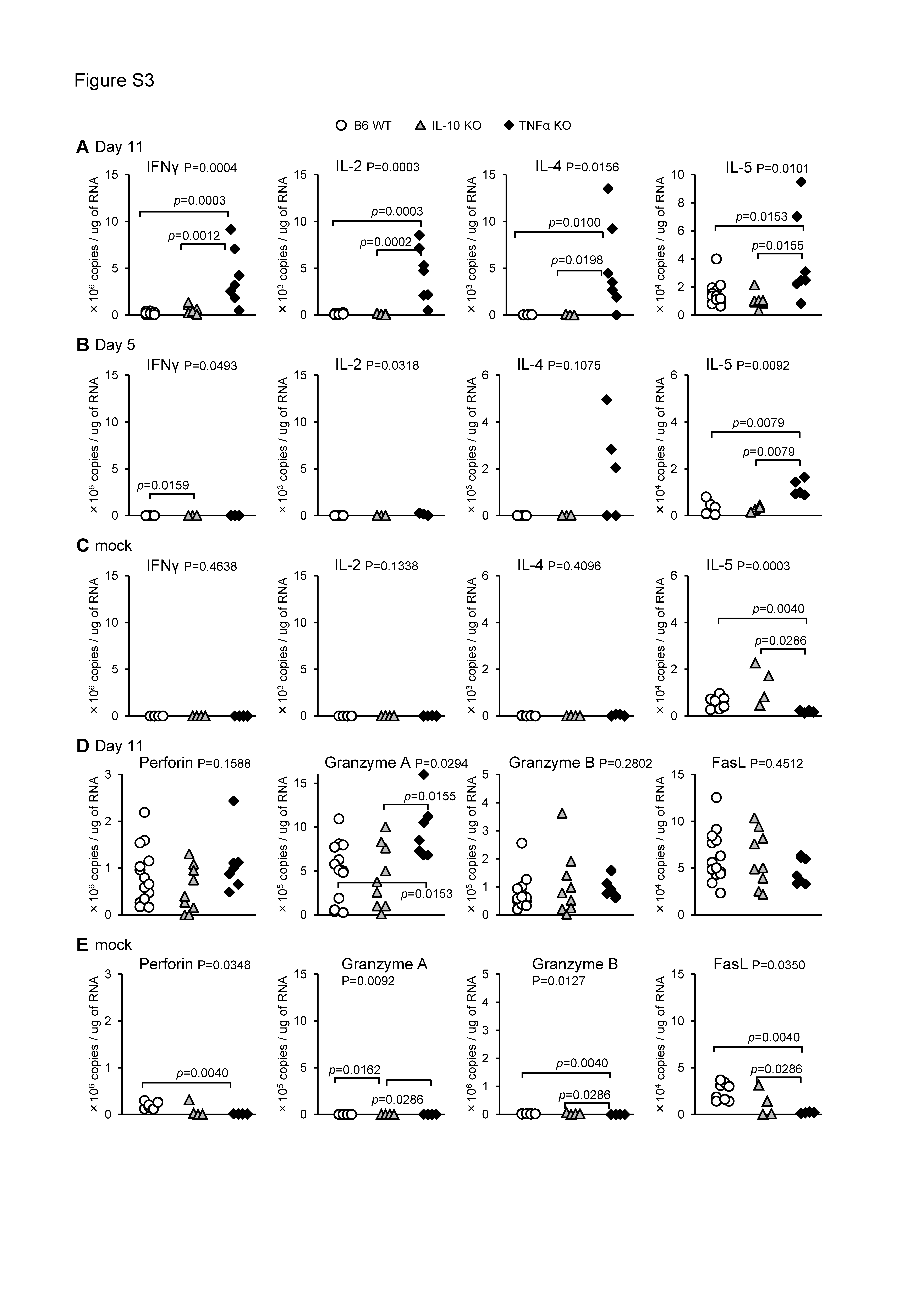

Supplement: Figure S3 — (A to C) mRNA levels of IFNγ, IL-2, IL-4, IL-5 quantified by real-time PCR in the brain cortex of WT, TNF-α and IL-10 mice infected with 104 pfu of JaOArS982 at 11 (A) and 5 (B) days pi and uninfected mice (C). (D and E) mRNA levels of perforin, granzyme A, granzyme B and FasL in the brain cortex of WT, TNF-α and IL-10 mice infected JaOArS982 at 11 days pi (D) and uninfected mice (E). (TIF) [file pone.0071643.s003.tif]

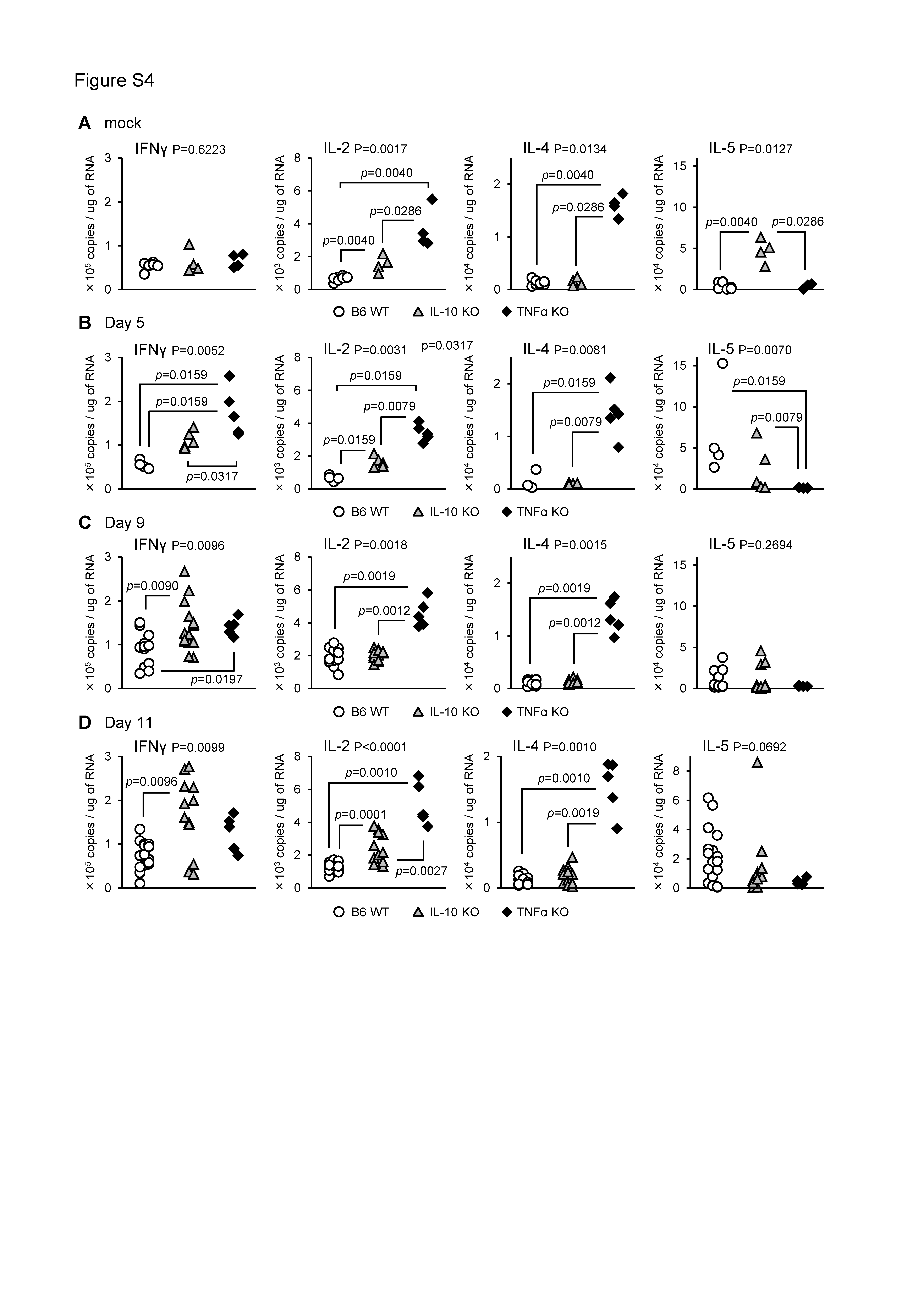

Supplement: Figure S4 — (A) mRNA levels of IFNγ, IL-2, IL-4, IL-5 quantified by real-time PCR in the spleen of WT, TNF-α and IL-10 mice infected with mock (A) and 104 pfu of JaOArS982 at 5 (B), 9 (C) and 11 (D) days pi. P: Kruskal-Wallis test, p: Mann Whitney test. (TIF) [file pone.0071643.s004.tif]

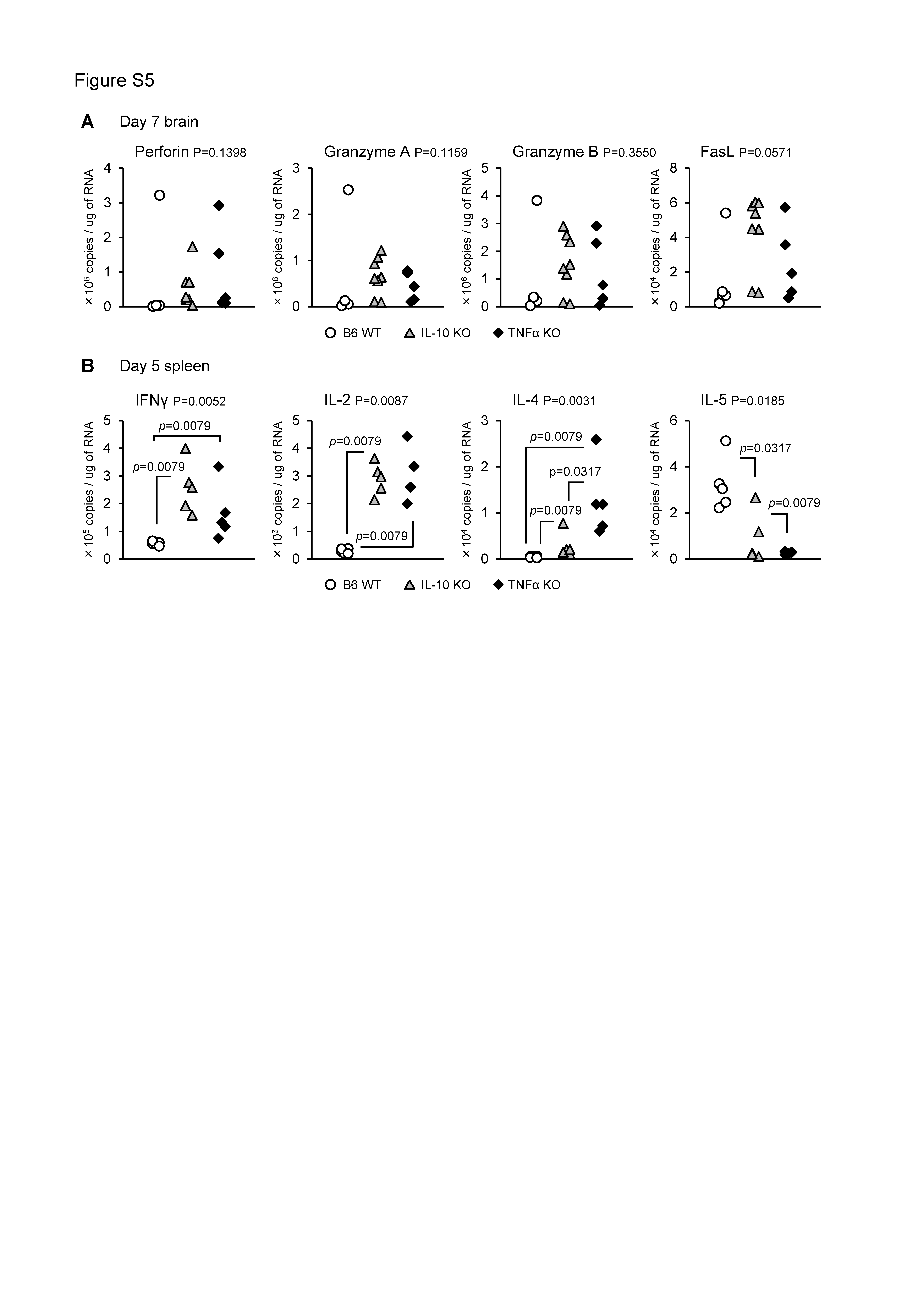

Supplement: Figure S5 — (A) mRNA levels of perforin, granzyme A, granzyme B and FasL in the brain cortex of WT, IL-10 and TNF-α mice at 7 days pi. P: Kruskal-Wallis test. (B) mRNA levels of IFNγ, IL-2, IL-4, IL-5 in the spleen of WT, IL-10 and TNF-α mice at 5 days pi. P: Kruskal-Wallis test, p: Mann Whitney test. (TIF) [file pone.0071643.s005.tif]
